# Genetic Interactions Between the Meiosis-Specific Cohesin Components, STAG3, REC8, and RAD21L

**DOI:** 10.1534/g3.116.029462

**Published:** 2016-04-16

**Authors:** Ayobami Ward, Jessica Hopkins, Matthew Mckay, Steve Murray, Philip W. Jordan

**Affiliations:** * Department of Biochemistry and Molecular Biology, Johns Hopkins University Bloomberg School of Public Health, Baltimore, Maryland 21205; † The Jackson Laboratory, Bar Harbor, Maine 04609

**Keywords:** cohesin, STAG3, synapsis, SMC, meiosis

## Abstract

Cohesin is an essential structural component of chromosomes that ensures accurate chromosome segregation during mitosis and meiosis. Previous studies have shown that there are cohesin complexes specific to meiosis, required to mediate homologous chromosome pairing, synapsis, recombination, and segregation. Meiosis-specific cohesin complexes consist of two structural maintenance of chromosomes proteins (SMC1α/SMC1β and SMC3), an α-kleisin protein (RAD21, RAD21L, or REC8), and a stromal antigen protein (STAG1, 2, or 3). STAG3 is exclusively expressed during meiosis, and is the predominant STAG protein component of cohesin complexes in primary spermatocytes from mouse, interacting directly with each α-kleisin subunit. REC8 and RAD21L are also meiosis-specific cohesin components. *Stag3* mutant spermatocytes arrest in early prophase (“zygotene-like” stage), displaying failed homolog synapsis and persistent DNA damage, as a result of unstable loading of cohesin onto the chromosome axes. Interestingly, *Rec8*, *Rad21L* double mutants resulted in an earlier “leptotene-like” arrest, accompanied by complete absence of STAG3 loading. To assess genetic interactions between STAG3 and α-kleisin subunits RAD21L and REC8, our lab generated *Stag3*, *Rad21L*, and *Stag3*, *Rec8* double knockout mice, and compared them to the *Rec8*, *Rad21L* double mutant. These double mutants are phenotypically distinct from one another, and more severe than each single knockout mutant with regards to chromosome axis formation, cohesin loading, and sister chromatid cohesion. The *Stag3*, *Rad21L*, and *Stag3*, *Rec8* double mutants both progress further into prophase I than the *Rec8*, *Rad21L* double mutant. Our genetic analysis demonstrates that cohesins containing STAG3 and REC8 are the main complex required for centromeric cohesion, and RAD21L cohesins are required for normal clustering of pericentromeric heterochromatin. Furthermore, the STAG3/REC8 and STAG3/RAD21L cohesins are the primary cohesins required for axis formation.

Formation of chromosomally normal gametes relies on accurate meiotic progression. During meiosis, replicated chromosomes undergo two consecutive rounds of chromosome segregation, namely meiosis I and meiosis II. Meiosis I is termed a “reductional” event because associated homologous chromosomes segregate from one another, whereas meiosis II is termed “equational” as sister chromatids segregate. To facilitate accurate chromosome segregation during meiosis I homologous chromosomes must pair, recombine and synapse. Inability to efficiently link homologs prior to their segregation leads to cell cycle arrest, apoptosis, or gamete aneuploidy.

Since sequencing technologies for screening human mutations have become more cost effective, there has been an influx of mutations identified to cause male and female infertility (Lin and Matzuk 2014). Recently, mutation of *Stag3* was found to cause premature ovarian failure (Caburet *et al.* 2014). Analysis of mouse models of the *Stag3* mutant showed that the mutation results in both male and female infertility, due to a failure to pair and synapse homologous chromosomes, which resulted in cell cycle arrest and apoptosis (Caburet *et al.* 2014; Fukuda *et al.* 2014; Hopkins *et al.* 2014; Llano *et al.* 2014; Winters *et al.* 2014).

Stromal antigen protein, STAG3, is a meiosis-specific component of cohesin (Prieto *et al.* 2001). Cohesin is best known for its role in maintaining sister chromatid cohesion prior to the metaphase to anaphase transition during mitosis (Michaelis *et al.* 1997). Cohesin is comprised of two structural maintenance of chromosome proteins (SMC3, and SMC1α or SMC1β), an α-kleisin subunit (RAD21, RAD21L, or REC8), and a stromal antigen protein (STAG1, 2, or 3) (Gutiérrez-Caballero *et al.* 2011; Ishiguro *et al.* 2011; Lee and Hirano 2011). Structural and interaction studies have demonstrated that the SMC1 and SMC3 proteins interact with one another at a central hinge domain, and then fold back on themselves through two large coiled-coil domains resulting in the juxtaposition of their own N and C termini, which are called the head domains. The head domains of SMC1 and SMC3 are bridged by an α-kleisin subunit (Nasmyth and Haering 2005). The cohesin complex also comprises one of the STAG proteins, which interact with the α-kleisin. Based on *in vitro* and *in vivo* studies using budding yeast, it has been proposed that the STAG protein is required either for cohesin binding to chromosomes or for the stability of binding to chromosomes (Orgil *et al.* 2015; Rowland *et al.* 2009; Sutani *et al.* 2009; Tóth *et al.* 1999). Using HeLa cells, it has been shown that STAG2 is the predominant cohesin component in mitotic cells, and is required for cohesion along chromosome arms and centromeres, whereas STAG1 is required for cohesion at telomeres (Hauf *et al.* 2005; Canudas and Smith 2009; Holzmann *et al.* 2011; Sumara *et al.* 2000; Losada *et al.* 2000).

During meiosis, cohesin complexes are important for chromosome pairing and are thought to be structurally intrinsic to the formation of axes between sister chromatids during prophase I (Heidmann *et al.* 2004; Ishiguro *et al.* 2014; Klein *et al.* 1999; Llano *et al.* 2012; Manheim and McKim 2003; Mito *et al.* 2003; Pasierbek *et al.* 2003). At the preleptotene stage, telomeres become attached to the nuclear periphery, and initial chromosome pairing events are facilitated by rapid chromosome movements (Boateng *et al.* 2013; Scherthan *et al.* 1996; Shibuya *et al.* 2014). Meiosis-specific cohesins are required for stable telomere attachment to the nuclear envelope (Adelfalk *et al.* 2009; Herrán *et al.* 2011; Shibuya *et al.* 2014). Mouse chromosomes are telocentric, and the pericentromeric heterochromatin regions assemble in clusters during preleptotene to form “chromocenters”, which are also thought to be required for chromosome pairing (Ishiguro *et al.* 2011; Scherthan *et al.* 1996; Shibuya *et al.* 2014). At the leptotene stage of meiotic prophase I, DNA double-strand breaks (DSBs) are catalyzed by the meiosis-specific topoisomerase II-like enzyme, SPO11 (Keeney *et al.* 1997). These DSBs stimulate the ataxia telangiectasia mutated (ATM) and Rad3-related (ATR) kinases to signal a DNA damage response, which results in phosphorylation of histone H2AFX (γH2AX) and recruitment of DNA repair proteins (Bellani *et al.* 2005; Bolcun-Filas and Schimenti 2012; Royo *et al.* 2013). Physical associations between homologous chromosomes are required to facilitate DNA repair. Homolog interactions are stabilized during the zygotene stage of prophase I, and dynamic movement of the chromosomes at this stage results in telomere clustering (“bouquet formation”) at the nuclear periphery. DNA repair and associations between homologs are stabilized by the formation of a tripartite proteinaceous structure known as the synaptonemal complex (SC) (Handel and Schimenti 2010). Cohesin complexes together with other axial proteins such as the SC proteins SYCP2 and SYCP3, form the two lateral elements of the SC, which are bridged together by the transverse filament protein, SYCP1, thus stimulating homolog synapsis (Moens *et al.* 1987; Offenberg *et al.* 1998; Yuan *et al.* 2000). At the pachytene stage of prophase I, homologs are fully synapsed and DNA repair is largely completed (Bolcun-Filas and Schimenti 2012; Handel and Schimenti 2010). Errors during chromosome pairing, DNA repair or synapsis generally result in meiotic cell cycle arrest and apoptosis.

Cohesin complexes are required for a number of different roles during meiosis I, including telomere attachment to the nuclear periphery, telomere maintenance, chromosome pairing, chromosome synapsis and maintenance of sister chromatid cohesion (Adelfalk *et al.* 2009; Bannister *et al.* 2004; Biswas *et al.* 2013; Fukuda *et al.* 2014; Heidmann *et al.* 2004; Herrán *et al.* 2011; Hopkins *et al.* 2014; Ishiguro *et al.* 2014; Khetani and Bickel 2007; Klein *et al.* 1999; Llano *et al.* 2012, 2014; Manheim and McKim 2003; Mito *et al.* 2003; Novak *et al.* 2008; Pasierbek *et al.* 2003; Revenkova *et al.* 2004; Winters *et al.* 2014; Xu *et al.* 2005). How does cohesin facilitate so many processes during meiosis? In part, this can be explained by the fact that there is one SMC1 protein (SMC1β), two α-kleisins (RAD21L and REC8), and one STAG protein (STAG3) that are specifically expressed in meiosis. Previous interaction studies indicate that there are at least four to six meiosis-specific forms of cohesin (Gutiérrez-Caballero *et al.* 2011; Ishiguro *et al.* 2011; Lee and Hirano 2011; Winters *et al.* 2014). In addition, the cohesin complexes expressed during mitosis are also present during meiosis (Gutiérrez-Caballero *et al.* 2011; Ishiguro *et al.* 2011; Lee and Hirano 2011). These interaction studies suggest that STAG3 is the predominant STAG subunit of cohesins during mouse spermatogenesis (Gutiérrez-Caballero *et al.* 2011; Ishiguro *et al.* 2011; Lee and Hirano 2011).

In order to delineate function, mouse mutants for all meiosis-specific subunits of cohesin have been characterized. In males, mutation of *Smc1β* results in a pachytene-like arrest where most homologous chromosomes synapse, but the SYCP3 length is reduced to ∼50%, and the sex chromosomes fail to pair (Novak *et al.* 2008; Revenkova *et al.* 2004). Although mutation of *Smc1β* in female mice causes a similar phenotype with regard to SYCP3 length, the oocytes progress through meiosis; however, loss of chromatid cohesion results in aneuploidy that is exacerbated with age (Revenkova *et al.* 2004; Hodges *et al.* 2005). Additionally, heterozygous mutation of *Smc1β* results in erroneous synapsis, decreased levels of crossover recombination and aneuploidy (Murdoch *et al.* 2013). The phenotypes derived from mutation of *Rad21l* are also sexually dimorphic (Herrán *et al.* 2011). *Rad21l* mutant spermatocytes arrest at a zygotene-like stage that is characterized by incomplete synapsis between homologs, synapsis between nonhomologous chromosomes, and an inability to repair meiotic DSBs. In contrast, oocytes from *Rad21l* mutants display close to normal synapsis between homologs, and progression through meiosis is observed. However, the female *Rad21l* mutants are subfertile, and demonstrate premature ovarian failure. Mutation of the gene encoding the other meiosis-specific kleisin subunit, *Rec8*, also results in a zygotene-like arrest; however, features of this arrest are distinct from the *Rad21l* mutant (Bannister *et al.* 2004; Xu *et al.* 2005). *Rec8* mutation results in aberrant synapsis between sister chromatids, a partial defect in DSB repair, and precocious loss of centromeric cohesion. This zygotene-like arrest is also observed in *Rec8* mutant oocytes. Furthermore, heterozygous mutation of *Rec8* results in a higher frequency of sister chromatid cohesion loss in MI oocytes (Murdoch *et al.* 2013). Mutation of the meiosis-specific STAG gene, *Stag3*, resulted in the most severe single-gene phenotype reported, and harbors characteristics of the *Rad21l* and *Rec8* mutants (Caburet *et al.* 2014; Fukuda *et al.* 2014; Hopkins *et al.* 2014; Llano *et al.* 2014; Winters *et al.* 2014). Both male and female gametocytes arrest at a zygotene-like stage with SYCP3 stretches that are ∼33% of the normal length, and synapsis between sister chromatids is observed. In addition, DSBs are not repaired, and cohesion between sister chromatid centromeres is lost prematurely. Although interaction data have demonstrated that STAG3 is the predominant STAG protein component of cohesin complexes in primary spermatocytes from mouse, we observed limited amounts of meiosis-specific cohesin components colocalizing to the SYCP3 stretches (Hopkins *et al.* 2014). This led us to hypothesize that STAG3 is required for stabilizing the majority of the meiosis-specific cohesin complexes to chromosome axes, and that STAG1 and STAG2 may be able to partially compensate. Interestingly, the combination of *Rad21l* and *Rec8* mutations results in a leptotene-like stage arrest, with a small number of short SYCP3 signals that consisted only low levels of mitotic cohesin components, RAD21 and SMC3. In this study, we combined mutations of *Stag3* with *Rad21l* and *Rec8* mutations, respectively, and compared them side-by-side with the single mutants, as well as the *Rad21l*, *Rec8* double mutant. We determined that the *Stag3*, *Rad21l* and *Stag3*, *Rec8* double mutant mice exhibit more pronounced meiotic defects than the *Stag3* single mutant, including a decrease in SYCP3 signal lengths, an increase in SYCP3 number, enhanced centromere cohesion defects, and decreased levels of SYCP3-associated cohesin. In addition, these double mutants display unique phenotypes from one another. We also determined that the phenotypes observed for these double knockout mice are distinct from the phenotype presented by the *Rad21l*, *Rec8* double mutant, which has lower number of short SYCP3 stretches and virtually no cohesin loading. We demonstrate that STAG3/REC8 cohesins are critical for sister chromatid cohesion, and that RAD21L cohesins are uniquely required for normal pericentromeric heterochromatin clustering events. Our study also suggests that STAG3/REC8 and STAG3/RAD21L cohesins are critical for the formation of chromosomal axes during meiotic prophase.

## Materials and Methods

### Ethics statement

All mice were bred by the investigators at The Jackson Laboratory (JAX, Bar Harbor, ME) and Johns Hopkins University (JHU, Baltimore, MD) under standard conditions in accordance with the National Institutes of Health (NIH) and US Department of Agriculture criteria, and protocols for their care and use were approved by the Institutional Animal Care and Use Committees (IACUC) of both JAX and JHU.

### Mice

The two *Stag3* mutations used in this study have been described previously (Hopkins *et al.* 2014). Briefly, the *Stag3^OV^* allele was created by integration of the SB-cHS4core-SB-Tyro-WPRE-FUGW lentiposon transgene (LV2229) in intron 8 of *Stag3* (https://www.jax.org/strain/017424) in an FVB background. The *Stag3^JAX^* allele was obtained by targeting C57BL/6N-derived JM8.N4 embryonic stem (ES) cells with a *β-galactosidase*-containing cassette that generated a knockout first reporter allele for *Stag3* that harbored a floxed exon 5 (http://www.mousephenotype.org/data/alleles/project_id?ikmc_project_id=22907). The corresponding mice were bred to B6N.Cg-Tg(Sox2-cre)1Amc/J mice to excise the floxed neomycin cassette and exon 5. The resulting mice [B6N(Cg)-Stag3tm1b(KOMP)Wtsi/2J] were used in this study. The *Rec8* (B6;129S4-*Rec8^mei8^*) mutant mice used in our study has previously been described (Bannister *et al.* 2004). Briefly, the *Rec8^Mei8^* allele contains a C > T nonsense mutation corresponding to the terminal amino acid of exon 6, creating a premature translational stop at amino acid 154 of 591 and eliminating exons 7 through 20. The *Rad21l^JAX^* allele was obtained using C57BL/6N-derived JM8.N4 ES cells that were targeted with a *β-galactosidase*-containing cassette. This generated a knockout first reporter allele for *Rad21l* that harbored a floxed exon 3. These ES cells were sourced from the International Knockout Mouse Consortium (Skarnes *et al.* 2011), http://www.mousephenotype.org/data/genes/MGI:3652039). As part of the KOMP2 program (http://commonfund.nih.gov/KOMP2/), these ES cells were injected into B6(Cg)-Tyrc-2J/J blastocysts. The resulting chimeric males were bred to C57BL/6NJ females, and then to B6N.Cg-Tg(Sox2-cre)1Amc/J mice to excise the floxed neomycin cassette and exon 3 (Supplemental Material, Figure S1). Offspring were bred to C57BL/6NJ mice to remove the cre-expressing transgene resulting in the B6N(Cg)-Rad21ltm1b(KOMP)Wtsi/2J strain used in this study. Single heterozygous mutants of each gene were bred together to create F1 double heterozygous mutants. These were subsequently bred to create homozygous double mutants. At least three mice for each double mutant were assessed, and compared with littermate controls that were either homozygote for a single gene or heterozygote for both genes.

### Mouse germ-cell isolation

Germ cells were isolated from 14- to 15-d-old male mice, which are enriched for midprophase spermatocytes. Isolation of mixed germ cells from testes was performed using techniques previously described (Bellve 1993; La Salle *et al.* 2009). Mice were killed via cervical dislocation, testes were obtained, and the tunica albicans removed. Using forceps, the testis tubules were shredded in Krebs-Ringer Bicarbonate Buffer (KRB) supplemented with protease inhibitor (PI) cocktail solution to liberate germ cells. The cell suspensions were filtered through a 0.8 µm Nitex mesh.

### Spread chromatin analyses

Germ cell chromatin spreads were prepared as previously described (Gómez *et al.* 2013; Jordan *et al.* 2012). Isolated germ cells were centrifuged at 5800 × *g* for 5 min. Cells were resuspended in 0.1 M sucrose solution, and dropped onto slides prewet with 1% PFA, 0.05% Triton-X solution. Slides were incubated at room temperature for 2.5 hr, and washed with 1× PBS, 0.4% Kodak Photoflo solution. Slides were air-dried, and then incubated for 30 min in prehybridization solution. Primary antibodies and the dilution used are presented in Table S1. Secondary antibodies against human, rabbit, rat, mouse, and goat IgG, and conjugated to Alexa 488, 568, or 633 (Life Technologies) were used at 1:500 dilution. Chromatin spreads were mounted in Vectashield + DAPI medium (Vector Laboratories).

### Microscopy and image analyses

Nuclear spread images were captured using a Zeiss CellObserver Z1 linked to an ORCA-Flash 4.0 CMOS camera (Hamamatsu), and analyzed with the Zeiss ZEN 2012 blue edition image software including foci and length measurement capabilities. ZEN 2012 image software was also used to determine and subtract background from each image then calculate the Manders’ colocalization/overlap coefficient (MCC) for the signal obtained for each cohesin component within the SYCP3 axes (Dunn *et al.* 2011). Image J was used to count chromocenters. Photoshop (Adobe) was used to prepare figure images.

### Data availability

The authors state that all data necessary for confirming the conclusions presented in the article are represented fully within the article.

## Results

### Combining the Stag3 mutation With Rec8 or Rad21l mutations result in decreased SYCP3 length and increased SYCP3 number

We compared single homozygous mutants for *Stag3*, *Rad21l*, and *Rec8*, together with the three combinations of homozygous double mutants (*i.e.*, *Stag3*, *Rad21l*; *Stag3*, *Rec8*, and *Rad21l*, *Rec8*). Each single and double homozygous mutant harbored small testes (Figure S2A). Analysis of γH2AX signal on meiotic chromatin spreads indicated that each mutant was proficient in initiating a DNA damage response; however, they were unable to repair the DSBs formed (Figure S2B). The most advanced meiotic stages observed for the *Rad21l* and *Rec8* single mutants displayed γH2AX enriched in concentrated patches, indicating that some DNA repair may have occurred. In contrast, the γH2AX signal was diffuse throughout the chromatin in the *Stag3* mutant. Each of the three double knockout combinations displayed similar diffuse γH2AX signal.

**Figure 1:**
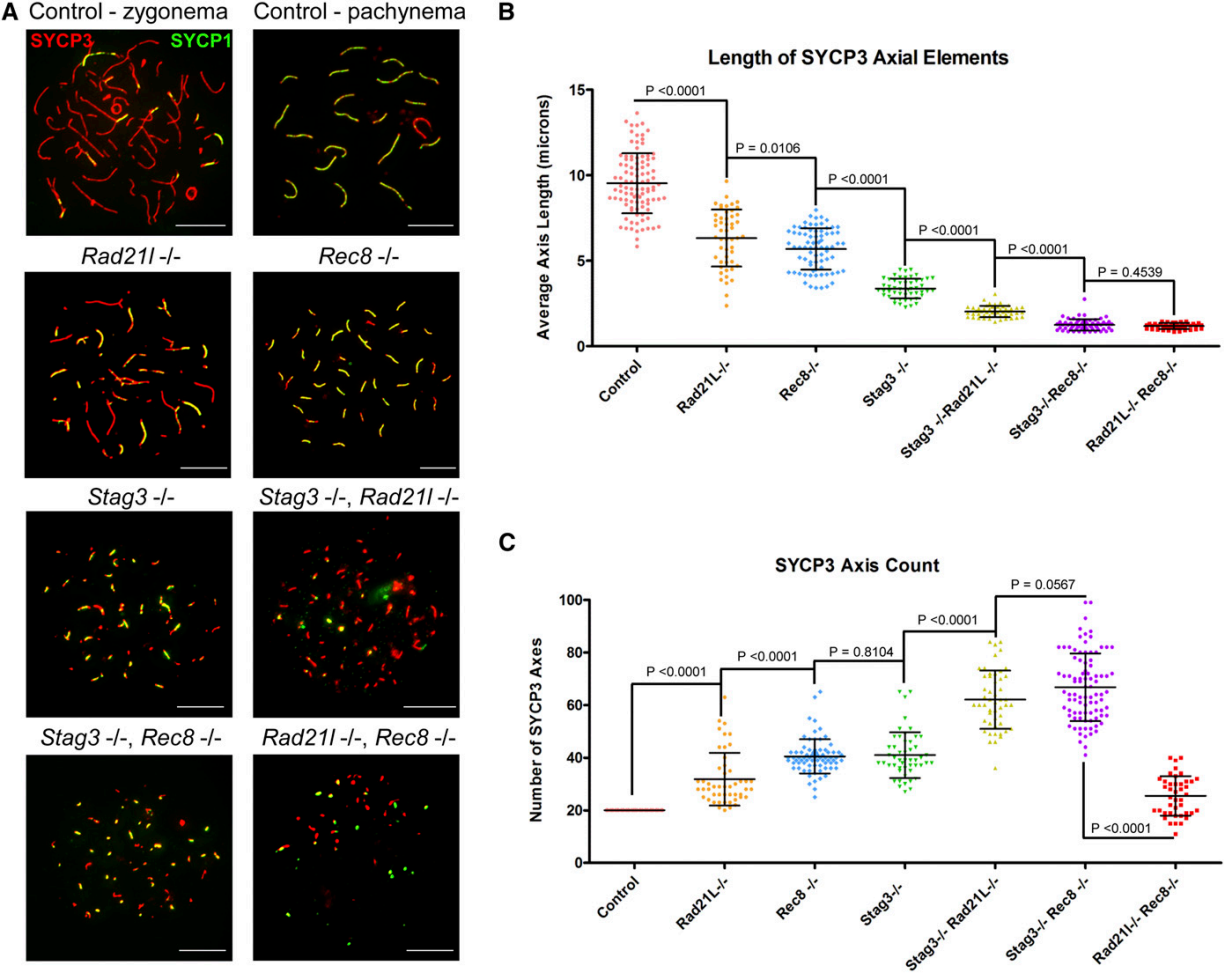
Combining the *Stag3* mutation with *Rec8* or *Rad21l* mutations result in decreased axis length and increased axis number. (A) Example chromatin spread preparations from purified testicular germ cells of control, *Rad21l*, *Rec8*, *Stag3* single mutants and the three possible double mutant combinations aged 15 d postpartum. Chromatin spreads were immunolabeled using antibodies against the SC lateral element protein SYCP3 (red), and the transverse filament of the central region of the SC SYCP1 (green). Zygotene and pachytene stages are depicted for control, and typical examples for each mutant are given. (B) Scatter dot-plot graph of the average SYCP3 length per spermatocyte chromatin spread. (C) Scatter dot-plot graph of the number of SYCP3 linear stretches per spermatocyte chromatin spread. Mean and SD of the columns of each graph are represented by the black bars, and *P* values are given for indicated comparisons (Mann–Whitney, two-tailed), significant differences were defined when the *P* value was < 0.05, otherwise it was considered not significant. Experiments were performed using four separate littermate pairs of mutant and control mice. Scale bars = 10 µm. Images in (A), and data in (B) and (C) are of spermatocytes carrying the *Stag3^OV^* mutant allele, but similar phenotypes were observed for spermatocytes with the *Stag3^JAX^* mutant allele (Figure S3).

To assess the meiotic defects of each mutant, we analyzed the formation of chromosome axes using immunofluorescence microscopy of spread chromatin (Figure 1A). We staged the progression of prophase I using antibodies against axial/lateral element, SYCP3, and the central region protein SYCP1. The most advanced stage was assigned based on the extent of SYCP1 staining, which signifies chromosome synapsis. We used wild-type pachytene stage as a reference of SYCP3 length observed during normal meiotic progression. Based on the 20 synapsed SYCP3 axes, we measured an average length of 9.46 µm ± 1.71 µm per axis (*N* = 100 nuclei). Each cohesin mutant resulted in arrest at a zygotene-like stage. However, the different mutants showed distinct aberrant features with regards to SYCP3 length and number (Figure 1, B and C). The *Rad21l* mutant exhibited partially synapsed axes that were fragmented; the SYCP3 length averaged 6.29 µm ± 0.96 µm, and the number of SYCP3 stretches averaged 31.7 ± 9.9 (*N* = 50 nuclei). The *Rec8* mutants also displayed a decrease in SYCP3 length, averaging 5.63 µm ± 1.24 µm (*N* = 75 nuclei). However, axis fragmentation was not commonly observed in the *Rec8* mutant chromatin spreads. The number of SYCP3 stretches observed in *Rec8* mutant chromatin spreads was 40.5 ± 8, which corresponds with previous work that showed synapsis occurs between sister chromatids (Bannister *et al.* 2004; Xu *et al.* 2005). Chromatin spreads from the *Stag3* mutant primary spermatocytes displayed ∼40–50% shorter SYCP3 stretches than either the *Rad21l* and *Rec8* mutants, 3.32 µm ± 0.61 µm (*N* = 50 nuclei). The SYCP3 count is similar to the *Rec8* mutant, averaging 41 ± 8.5 axes. The extensive synapsis observed in *Stag3* mutant chromatin spreads has been shown to be primarily between sister chromatids (Fukuda *et al.* 2014; Hopkins *et al.* 2014).

Combination of the *Stag3* mutation with the *Rad21l* and *Rec8* mutations resulted in further decreases in SYCP3 length (Figure 1). The average SYCP3 lengths quantified from the *Stag3*, *Rad21l* double mutant (2.01 µm ± 0.32 µm, *N* = 50 nuclei) were longer than those observed for the *Stag3*, *Rec8* double mutant (1.21 µm ± 0.35 µm, *N* = 65 nuclei). The SYCP3 counts for these double mutants were greater than the single mutations, the *Stag3*, *Rad21l* double mutant having an average of 61.8 ± 11.1, and the *Stag3*, *Rec8* double mutant averaging 66.6 ± 12.8 SYCP3 stretches per chromatin spread. Additionally, the amount of SYCP1 signal observed on axes from the *Stag3*, *Rec8* double mutant was much more extensive than that observed for the *Stag3*, *Rad21l* double mutant (Figure 1A). We also obtained data that were consistent with these observations by using an independently derived *Stag3* mutation, which was combined with the *Rad21l* and *Rec8* mutations (Figure S3). The *Rad21l*, *Rec8* double mutant also displayed short SYCP3 length, averaging 1.14 µm ± 0.19 µm (*N* = 50 nuclei). However, the number of SYCP3 signals was reduced compared to the other double mutants, with an average of 25.3 ± 7.4 signals per chromatin spread. Low levels of SYCP1 signal that colocalized with SYCP3 were observed on the *Rad21l*, *Rec8* double mutant chromatin spreads. We also observed SYCP1-only positive foci in *Rad21l*, *Rec8* double knockout chromosome spreads, which may be due to inappropriate loading of SYCP1 onto the chromatin or nonspecific staining from the antibody.

### Compensation of centromeric cohesion in the absence of REC8

**Figure 2:**
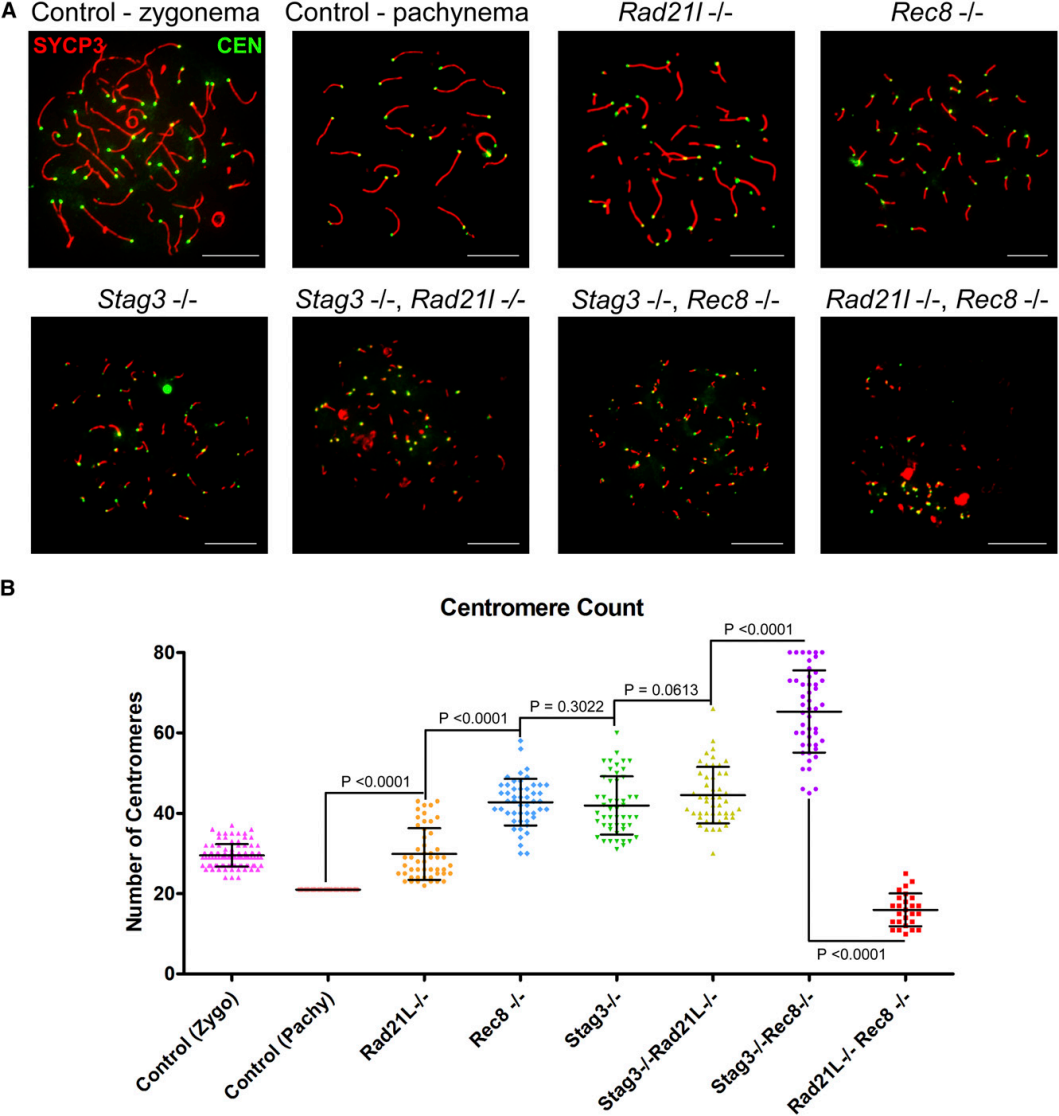
STAG3 maintains centromere cohesion, which is primarily mediated by REC8-containing cohesin complexes. (A) Example chromatin spread preparations from purified testicular germ cells of control, *Rad21l*, *Rec8*, *Stag3* single mutants, and the three possible double mutant combinations aged 15 d postpartum. Chromatin spreads were immunolabeled using antibodies against the SC lateral element protein SYCP3 (red), and the CEN anti-centromere autoantibody (green). Zygotene and pachytene stages are depicted for control, and typical examples for each mutant are given. (B) Scatter dot-plot graph of the average number of centromere signals per spermatocyte chromatin spread. Mean and SD of the columns of each graph are represented by the black bars, and *P* values are given for indicated comparisons (Mann–Whitney, two-tailed), significant differences were defined when the *P* value was < 0.05, otherwise it was considered not significant. Experiments were performed using four separate littermate pairs of mutant and control mice. Scale bars = 10 µm. Images in (A), and data in (B) and (C) are of spermatocytes carrying the *Stag3^OV^* mutant allele, but similar phenotypes were observed for spermatocytes with the *Stag3^JAX^* mutant allele (Figure S4).

Previous work has demonstrated that REC8-containing cohesins are the primary source of centromeric cohesion between sister chromatids (Herrán *et al.* 2011; Hopkins *et al.* 2014). However, other cohesin complexes may contribute. To assess this further, we counted centromere signals in chromatin spread preparations of the cohesin mutants, using an anti-centromere autoantibody (CEN; also known as ACA and CREST, Figure 2). We used wild-type littermate controls as a reference for centromere numbers during meiotic progression. Assessment of chromatin spread preparations from wild-type spermatocytes demonstrated that, at late zygotene stages, there are an average of 29.5 ± 6 (*N* = 100 nuclei) centromere signals, and this decreased by pachytene stage to 21 (*N* = 50 nuclei) centromere signals, corresponding to complete synapsis of homologous chromosomes. The *Rad21l* single mutant showed similar centromere numbers as wild-type spermatocytes during the late zygotene stage, with an average of 29.8 ± 6.6 (*N* = 50 nuclei) centromeres per nuclei. On the other hand, both the *Rec8* and *Stag3* mutant showed an increase in centromere signal, averaging 42.6 ± 5.9 and 42.4 ± 6.5 centromere counts respectively (*N* = 50 and 60 nuclei, respectively). At this stage, mouse spermatocytes contain 40 pairs of sister chromatids; however, a majority of the *Rec8* (64%) and *Stag3* (60%) mutant chromatin spreads have a centromere count of greater than 40. These observations are consistent with a role in maintaining centromeric cohesion between sister chromatids.

Combination of the *Stag3* mutation with the *Rad21l* mutation resulted in a very similar phenotype to the *Stag3* single mutant with regards to centromere signal (44.3 ± 6.9, *N* = 50 nuclei). This indicates that the RAD21L-containing cohesin complexes do not contribute significantly to cohesion between sister centromeres in *Stag3* mutants. In contrast the *Stag3*, *Rec8* double mutant showed an increase in centromere numbers compared to both of the single mutants, averaging 65.2 ± 10.1 centromeres per chromatin spread. This intriguing observation leads us to speculate that there is an additional or compensatory mechanism that partially maintains centromeric cohesion in the *Rec8* mutants, which is facilitated by the presence of STAG3. These data were consistent with the observations we made using an independently derived *Stag3* mutation, which was combined with the *Rad21l* and *Rec8* mutations (Figure S4). Centromere counts of chromatin spreads from the *Rad21l*, *Rec8* double mutant showed that there were decreased numbers of centromere signal compared to each of the other mutant combinations, and the wild-type control (16.3 ± 3.6 centromere signals, *N* = 50 nuclei). This is consistent with the reduced number of chromosome axes we observed for this mutant (Figure 1C), and suggests that centromeres are clustered together at the point of meiotic arrest.

### RAD21L and REC8 cohesins are differentially required for regulating pericentromeric heterochromatin clustering

**Figure 3:**
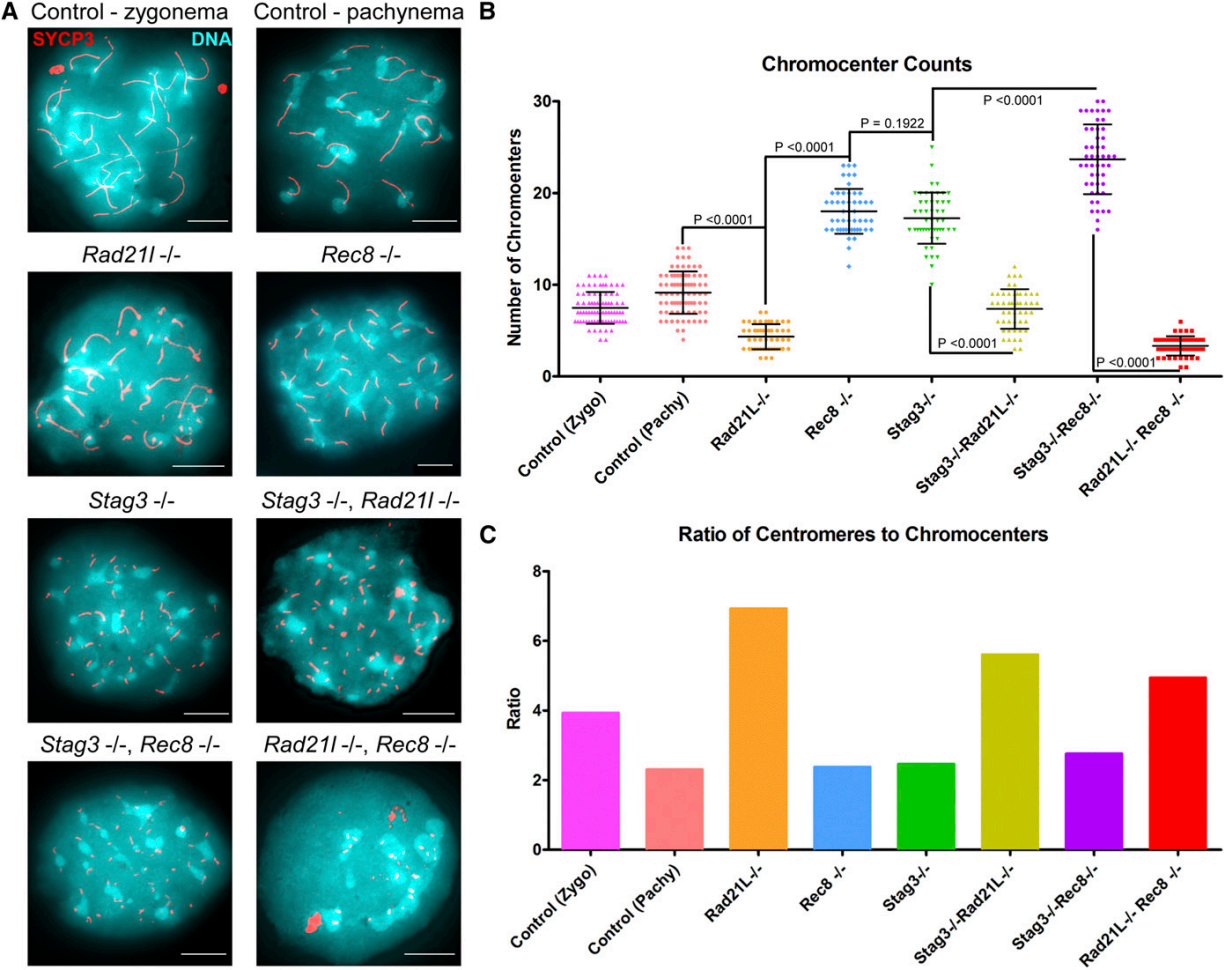
RAD21L and REC8 cohesins are differentially required for regulating pericentromeric heterochromatin clustering. (A) Example chromatin spread preparations from purified testicular germ cells of control, *Rad21l*, *Rec8*, *Stag3* single mutants, and the three possible double mutant combinations aged 15 d postpartum. Chromatin spreads were stained with DAPI (turquoise, DNA), and immunolabeled using antibodies against the SC lateral element protein SYCP3 (red). Zygotene and pachytene stages are depicted for control, and typical examples for each mutant are given. (B) Scatter dot-plot graph of the average number of pericentromeric heterochomatin signals per spermatocyte chromatin spread. Mean and SD of the columns of each graph are represented by the black bars and *P* values are given for indicated comparisons (Mann–Whitney, two-tailed), significant differences were defined when the *P* value was < 0.05, otherwise it was considered not significant. Experiments were performed using four separate littermate pairs of mutant and control mice. Scale bars = 10 µm.

Telomeres anchor to the nuclear envelope during preleptotene, and rapid chromosome movements facilitate initial pairing of homologous chromosomes (Boateng *et al.* 2013; Scherthan *et al.* 1996). Meiosis-specific cohesins localize to the telomeres at this stage and are required for stable telomere anchoring to the nuclear periphery (Herrán *et al.* 2011; Ishiguro *et al.* 2014; Shibuya *et al.* 2014; Shibuya and Watanabe 2014). Mouse chromosomes are telocentric and STAG3, REC8, and RAD21L cohesins localize in the proximity of the telomeres and heterochromatin rich pericentromeric clusters (“chromocenters”) that form during preleptotene, and are required for chromosome pairing (Ishiguro *et al.* 2011; Shibuya and Watanabe 2014). We previously reported that mutation of *Stag3* resulted in increased numbers of pericentromeric clusters (Hopkins *et al.* 2014), and sought to determine whether this feature could further delineate the phenotype of these meiosis-specific cohesin mutants (Figure 3 and Figure S5). For wild-type we observed an average of 7.5 ± 1.9 and 9.1 ± 2.7 chromocenters during zygotene and pachytene stages respectively (*N* = 80 nuclei). Our analysis revealed that the *Rad21l* mutant had reduced numbers of chromocenters compared to wild type, averaging 4.3 ± 1.3 per chromatin spread (*N* = 50 nuclei). In contrast the *Rec8* and *Stag3* mutants show an increase of chromocenter number, averaging 17.9 ± 2.5 and 17.2 ± 2.9 chromocenters per chromatin spread respectively (*N* = 50 nuclei). Interestingly, the ratio of centromere signals per chromocenter is similar between wild type at pachytene stage, and the *Stag3* and *Rec8* mutants, whereas it is increased fourfold in the *Rad21l* mutants (Figure 3C).

Our analysis of chromatin spreads from the *Stag3*, *Rad21l* double mutant showed that the chromocenter number and ratio of centromere signals per chromocenter was intermediate between the low numbers observed for the *Rad21l* mutant, and higher numbers observed for the *Stag3* mutant (7.9 ± 2.0 chromocenters, *N* = 50 nuclei, Figure 3). However, the ratio of centromere signals to chromocenters was similar to the *Rad21l* single mutant (Figure 3C). The higher level of chromocenters observed for both *Stag3* and *Rec8* mutants was exacerbated when the mutations were combined, resulting in an average of 23.6 ± 3.8 chromocenters (*N* = 50 nuclei). However, the ratio of centromere signals per chromocenter was similar to wild type at pachytene stage and the *Stag3* and *Rec8* single mutants (Figure 3C). Combination of the two α-kleisin mutations resulted in chromocenter numbers similar to the data obtained for the *Rad21l* single mutant (3.3 ± 1.1 chromocenters, *N* = 50 nuclei). Due to the decreased number in centromere signals observed for the *Rad21l*, *Rec8* double mutant, which is likely due to an inability to distinguish each centromere, the ratio of centromeres per chromocenter was reduced compared to the data obtained for the *Rad21l* single mutant (Figure 3C). Taken together, our data suggest that cohesins containing RAD21L are required following chromocenter clustering.

### Different defects in cohesin loading in mutant spermatocytes

**Figure 4:**
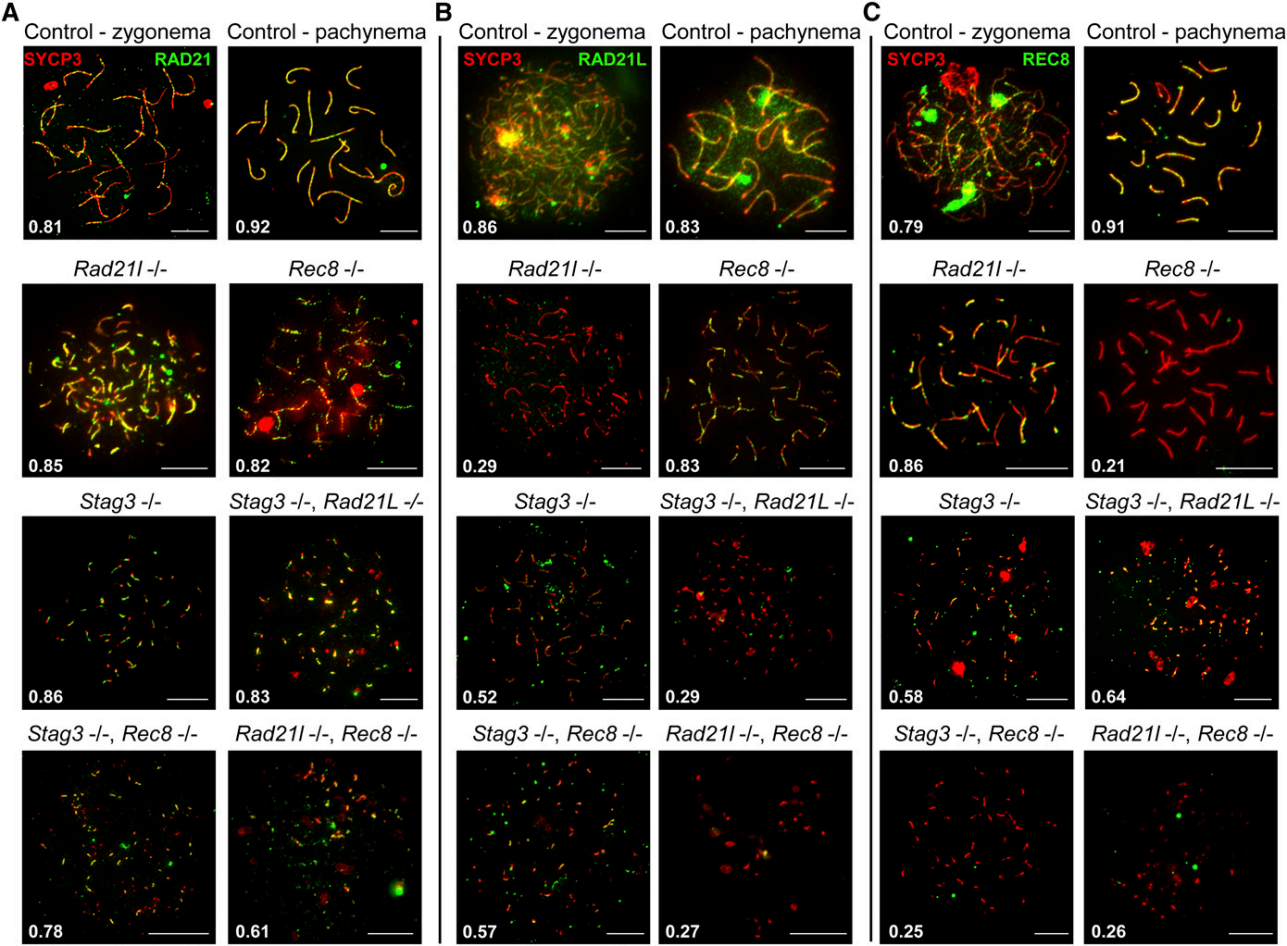
STAG3 is required for stable localization of RAD21L and REC8 cohesins at chromosome axes. (A–C) Example chromatin spread preparations from purified testicular germ cells of control, *Rad21l*, *Rec8*, *Stag3* single mutants, and the three possible double mutant combinations aged 15 d postpartum. Chromatin spreads were immunolabeled using antibodies against the SC lateral element protein SYCP3 (red) and either RAD21 (A), or RAD21L (B), or REC8 (C), all of which are shown in green. Zygotene and pachytene stages are depicted for control, and typical examples for each mutant are given. The numbers within each chromatin spread represent the average Manders’ colocalization/overlap coefficient for each cohesin component within the SYCP3 axes (*N* = 25 chromatin spreads per strain). Scale bars = 10 µm.

**Figure 5:**
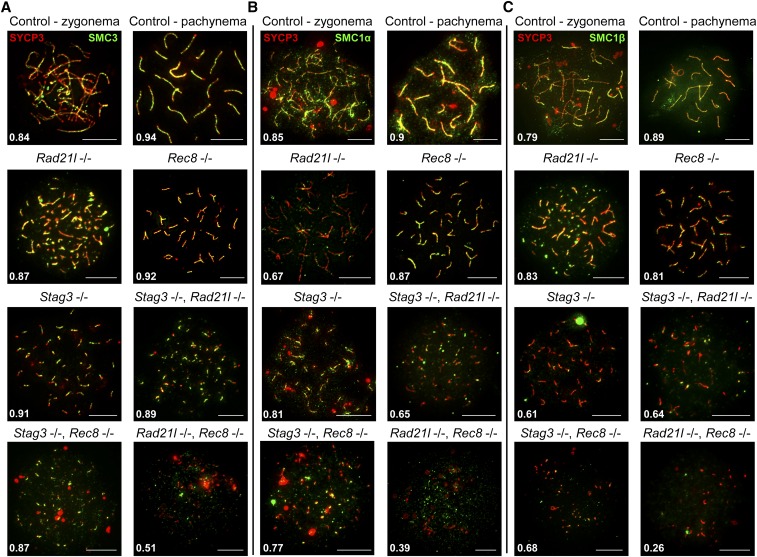
STAG3 is required for stable axial localization of meiosis-specific cohesins, but not the mitotic cohesins. (A–C) Example chromatin spread preparations from purified testicular germ cells of control, *Rad21l*, *Rec8*, *Stag3* single mutants, and the three possible double mutant combinations aged 15 d postpartum. Chromatin spreads were immunolabeled using antibodies against the SC lateral element protein SYCP3 (red), and either SMC3 (A), or SMC1α (B), or SMC1β (C), all of which are shown in green. Zygotene and pachytene stages are depicted for control, and typical examples for each mutant are given. The numbers within each chromatin spread represent the average Manders’ colocalization/overlap coefficient for each cohesin component within the SYCP3 axes (*N* = 25 chromatin spreads per strain). Scale bars = 10 µm.

As we observed varying phenotypes with regards to SYCP3 in each cohesin mutants, we hypothesized that cohesin colocalization with SYCP3 will also be differentially affected. We assessed the localization pattern observed for the mitotic (RAD21) and meiosis-specific (REC8 and RAD21L) α-kleisin subunits of cohesin, and the SMC components of cohesin, SMC3, SMC1α, and the meiosis-specific SMC1β (Figure 4 and Figure 5). Furthermore, we determined the average Manders’ colocalization/overlap coefficient (MCC) for the signal obtained for each cohesin component within the SYCP3 axes (Figure 4 and Figure 5; Dunn *et al.* 2011). Using littermate wild-type controls, we showed that each α-kleisin and SMC subunit localized along the chromosome axis during zygotene and pachytene stages (Figure 4 and Figure 5). As expected, we observed no signal for RAD21L in chromosome spreads from the *Rad21l* mutant, and similarly no signal for REC8 in chromosome spreads from the *Rec8* mutant. The *Rad21l* mutant did not display any major aberrancy with regard to localization of other cohesin components, with the exception of a slight decrease in SMC1α signal on SYCP3 stretches (Figure 5B). The decrease in SMC1α signal is consistent with what was previously observed reported (Herrán *et al.* 2011). The *Rec8* mutant did not show diminished colocalization between SYCP3 and any of the other cohesin components (Figure 4 and Figure 5). The *Stag3* mutant displayed decreased levels of colocalization with SYCP3 for all of the meiosis-specific cohesin components, RAD21L, REC8, and SMC1β (Figure 4, B and C, and Figure 5C), which is consistent with our previous observations (Hopkins *et al.* 2014). SMC3 and RAD21 colocalization with SYCP3 was not affected in any of the single mutants (Figure 4A and Figure 5A), which is consistent with a prior report (Llano *et al.* 2012).

The *Stag3*, *Rad21l* double mutant demonstrated diminished REC8, SMC1α, and SMC1β signal on SYCP3 stretches (Figure 4C, and Figure 5, B and C), which is a combination of the defects we observed in the two single mutants. Colocalization of cohesin with SYCP3 observed for the *Stag3*, *Rec8* double mutant was similar to the *Stag3* single mutant with RAD21L and SMC1β localization being diminished (Figure 4B and Figure 5C). The *Rad21l*, *Rec8* double mutant showed absence of RAD21L and REC8 immunostaining, and the signals for RAD21, SMC3, SMC1α, and SMC1β were greatly reduced (Figure 4 and Figure 5).

The data obtained for the single *Rad21l* and *Rec8* mutants, as well as in the presence of the *Stag3* mutation, indicates that REC8 and RAD21L loading onto the axes is independent from one another. As SMC1α localization is affected in the *Rad21l* mutant, we propose that RAD21L cohesins are primarily, but not exclusively, formed with SMC1α. Furthermore, STAG3 is required mainly for the stability of cohesins containing SMC1β. The decreased SYCP3 colocalization levels for all cohesin components observed for the *Rad21l*, *Rec8* double mutant indicates that all cohesins require the function of the two meiosis-specific kleisins in order to load onto chromosome axes.

We also assessed whether we could detect localization of STAG1 and STAG2 along the axes in wild-type and *Stag3* mutant chromatin spread preparations. However, we did not observe axial localization of either STAG1 or STAG2, which is consistent with a previous study that used the same antibodies (Figure S6; Fukuda *et al.* 2014).

## Discussion

### Cohesins and axis formation

Meiosis-specific cohesins are an integral component of the axes that form between chromosomes during prophase I. Based on interaction studies it has been shown that STAG3 is the primary STAG protein component of cohesin complexes during meiosis (Fukuda *et al.* 2014; Gutiérrez-Caballero *et al.* 2011; Ishiguro *et al.* 2011; Lee and Hirano 2011; Winters *et al.* 2014). We observed diminished, but not complete, loss of REC8 and RAD21L signal on the SYCP3 stretches in *Stag3* mutant spermatocytes. This suggests that STAG3 is required for stabilizing cohesin onto chromosome axes. Therefore, we sought to determine whether the *Stag3* mutant phenotype is exacerbated by combining the *Stag3* mutation with either *Rec8* or *Rad21l* mutations. Both double mutants display a further reduction in SYCP3 stretch length and increased stretch number. This supports the hypothesis that RAD21L- and REC8-containing cohesin rely on STAG3 for stabilization onto chromatin axes during meiotic prophase. The *Rad21*, *Rec8* double mutant spermatocytes arrest at an earlier stage of prophase compared to the *Stag3*, *Rad21l*, and *Stag3*, *Rec8* mutants. Considering that STAG3 does not localize to the short axes in the *Rad21l*, *Rec8* double knockout germ cells (Llano *et al.* 2012), it is reasonable to propose that combination of *Stag3*, *Rad21l*, and *Rec8* mutations as a triple knockout will result in a similar phenotype as the *Rad21l*, *Rec8* double knockout. The use of mutant analyses and protein localization studies to infer function is useful, but has obvious limitations, such as compensatory processes and detection thresholds. Additional analyses of unperturbed cells undergoing meiosis, and techniques that allow rapid, reversible protein target depletion studies can help verify these data and hypotheses.

Studies using budding yeast suggest that the STAG homolog, Scc3, is similarly required for stabilizing cohesin loading. For instance, a mutation in Scc3 that abolishes its ability to interact with the yeast α-kleisin subunit Scc1/Mcd1, results in decreased levels of Scc1/Mcd1 on the chromatin (Orgil *et al.* 2015). Furthermore, using an auxin-inducible degron (AID) system, it was shown that depletion of Scc3 resulted in partial loss of chromatin-bound Scc1/Mcd1 (Roig *et al.* 2014). In a converse experiment, AID-mediated depletion of Scc1/Mcd1 resulted in complete loss of Scc3. This mirrors the affect reported for the *Rec8* and *Rad21l* double mutant, which results in the complete loss of STAG3 localization (Llano *et al.* 2012), whereas mutation of *Stag3* results in depletion of REC8 and RAD21L.

The differences observed between the double knockout combinations with regards to axis morphology and cohesin loading may be explained by the presence or compensation of the mitotic STAG proteins, STAG1 and 2. One study has demonstrated that STAG2 localizes to meiotic chromosomes, and may participate in sister chromatid cohesion during diplotene stage of meiosis in mice (Prieto *et al.* 2002). Another study using antibodies developed by the investigators showed that STAG1 and STAG2 localize to the axes during meiotic prophase in wild-type and *Stag3* mutant spermatocytes (Llano *et al.* 2014). However, we did not observe STAG1 or STAG2 localization on the chromatin axes in wild-type or *Stag3* mutant spermatocyte chromatin spreads using commercially available antibodies, consistent with a previous report (Figure S6; Fukuda *et al.* 2014). Additional antibodies against STAG1 and STAG2 should be developed and made available to further test these contrasting observations. STAG1 and STAG2 proteins are expressed during spermatogenesis and were shown to interact with SMC1β, and these interactions are more prominent in *Stag3* mutant testis extracts (Winters *et al.* 2014). However, another report did not observe STAG1 or STAG2 interacting with SMC1β in wild-type or *Stag3* mutant extracts (Fukuda *et al.* 2014). More studies are required to determine whether the axis formation observed in *Stag3* mutants is due to the function of the other two STAG proteins. Development of conditional knockout mice will help determine the contribution of STAG1 and STAG2 during meiosis.

It is interesting to note that fission yeast expresses a meiosis-specific STAG protein, Rec11 (SA3), and it has recently been shown to be a phosphorylation target of the caesin kinase (CK) (Phadnis *et al.* 2015; Sakuno and Watanabe 2015). CK-mediated phosphorylation of Rec11 facilitates its interaction with lateral element proteins, which is required for proficient axis assembly. STAG3 is also phosphorylated, and future studies will determine whether its phosphorylation is important for normal axis formation (Fukuda *et al.* 2012; Jordan *et al.* 2012; Lee and Hirano 2011).

### REC8 and STAG3 are required for sister chromatid cohesion

Using okadaic acid to stimulate an artificial transition to metaphase, it was shown that *Rec8* and *Stag3* mutations result in loss of sister chromatid cohesion (Hopkins *et al.* 2014; Llano *et al.* 2014). This was not the case for *Rad21l* (Herrán *et al.* 2011). Here, we show that the *Stag3*, *Rec8* double mutant displays an additive effect on centromeric cohesion, again supporting the hypothesis that STAG3 is required to stabilize REC8 cohesins. In most cases, sister chromatid cohesin is not completely ablated, and this suggests that an additional cohesin complex or another mechanism is able to partially maintain sister chromatid cohesion. This is not likely to be cohesins containing RAD21L, as the *Stag3*, *Rad21l* double mutant does not show an increase in centromere counts compared to the *Stag3* mutant. The mitotic cohesin complex containing RAD21 may be responsible. Assessment of a conditional knockout mutant for *Rad21* will help resolve whether this is the case.

### Meiotic cohesins and pericentromeric heterochromatin clustering

Prior to meiotic entry, mouse telocentric chromosomes are observed in separate territories, and then at the preleptotene stage the pericentromeric heterochromatin and telomeres associate with the nuclear periphery (Scherthan *et al.* 1996). By late preleptotene stage, the pericentromeric heterochromatin forms clusters called “chromocenters” at the nuclear periphery. The chromocenters migrate to one position and form a single mass by the zygotene stage, which coincides with telomere “bouquet” formation. These events are known to be important for establishing homolog pairing, mediating DSB repair and synapsis. During the zygotene to pachytene stage transition, the pericentromeric heterochromatin mass separates into discrete chromocenters that remain attached to the nuclear periphery. STAG3, REC8, and RAD21L localize to the telomeres and chromocenters at preleptotene (Ishiguro *et al.* 2011; Lee and Hirano 2011; Shibuya *et al.* 2014). RAD21L localization in particular, overlaps well with the chromocenters (Ishiguro *et al.* 2011). The *Rad21l* mutant was reported to arrest at a zygotene-like stage with a telomere “bouquet” configuration (Ishiguro *et al.* 2014). Our results complement this as mutation of *Rad21l* results in a decrease number of chromocenters, and an increase in centromere number per chromocenter compared to the control. In contrast, *Stag3* and *Rec8* single mutants and double mutant result in double the amount of chromocenters, but similar numbers of centromeres per chromocenter compared to the control. The increase in chromocenters for these mutants is likely due to the synapsis between sister chromatids observed in these mutants (Fukuda *et al.* 2014; Hopkins *et al.* 2014; Xu *et al.* 2005). The *Stag3*, *Rad21l* and *Rad21l*, *Rec8* double mutant result in similar chromocenter counts to the *Rad21l* single mutant. RAD21L localizes specifically to the chromocenters during the preleptotene/early leptotene stage, whereas REC8 and STAG3 localize as punctate signals at the telomere (Lee and Hirano 2011; Shibuya *et al.* 2014). RAD21L was shown to be required for DSB-independent homolog recognition, this was not the case for REC8, and our results suggest that STAG3 is also not required (Ishiguro *et al.* 2014). As *Rad21l* mutant females display normal synapsis (Herrán *et al.* 2011), it is likely that this is a male-specific feature of mammalian meiosis, and further work addressing the differences between male and female meiosis is required.

### Conclusions

Our data further demonstrate that STAG3 is required for the stability of meiosis-specific cohesin complexes. Our genetic interaction analysis further delineates the unique properties of the REC8 and RAD21L cohesins. We show that the REC8-STAG3 cohesin complexes are the primary source of centromeric cohesion between sister chromatids. We propose that STAG3 is not essential for the DSB-independent chromosome pairing function of RAD21L. To support our findings, future work using biochemical approaches analyzing specific stages of meiosis, live cell imaging of spermatocyte cultures, and a degron-based protein depletion studies are needed.

## 

## Supplementary Material

Supplemental Material

## References

[bib1] Adelfalk C. Janschek J. Revenkova E. Blei C. Liebe B., 2009 Cohesin SMC1β protects telomeres in meiocytes. J. Cell Biol. 187: 185–199. 19841137 10.1083/jcb.200808016 PMC2768837

[bib2] Bannister L. A. Reinholdt L. G. Munroe R. J. Schimenti J. C., 2004 Positional cloning and characterization of mouse mei8, a disrupted allele of the meiotic cohesin Rec8. Genesis 40: 184–194. 15515002 10.1002/gene.20085

[bib3] Bellani M. A. Romanienko P. J. Cairatti D. A. Camerini-Otero R. D., 2005 SPO11 is required for sex-body formation, and Spo11 heterozygosity rescues the prophase arrest of Atm−/− spermatocytes. J. Cell Sci. 118: 3233–3245. 15998665 10.1242/jcs.02466

[bib4] Bellve A. R., 1993 Purification, culture, and fractionation of spermatogenic cells. Methods Enzymol. 225: 84–113. 8231890 10.1016/0076-6879(93)25009-q

[bib5] Biswas U. Wetzker C. Lange J. Christodoulou E. G. Seifert M., 2013 Meiotic cohesin SMC1β provides prophase I centromeric cohesion and is required for multiple synapsis-associated functions. PLoS Genet. 9: e1003985. 24385917 10.1371/journal.pgen.1003985 PMC3873225

[bib6] Boateng K. A. Bellani M. A. Gregoretti I. V. Pratto F. Camerini-Otero R. D., 2013 Homologous pairing preceding SPO11-mediated double-strand breaks in mice. Dev. Cell 24: 196–205. 23318132 10.1016/j.devcel.2012.12.002 PMC3562373

[bib7] Bolcun-Filas E. Schimenti J. C., 2012 Genetics of meiosis and recombination in mice. Int. Rev. Cell Mol. Biol. 298: 179–227. 22878107 10.1016/B978-0-12-394309-5.00005-5

[bib8] Caburet S. Arboleda V. A. Llano E. Overbeek P. A. Barbero J. L., 2014 Mutant cohesin in premature ovarian failure. N. Engl. J. Med. 370: 943–949. 24597867 10.1056/NEJMoa1309635 PMC4068824

[bib9] Canudas S. Smith S., 2009 Differential regulation of telomere and centromere cohesion by the Scc3 homologues SA1 and SA2, respectively, in human cells. J. Cell Biol. 187: 165–173. 19822671 10.1083/jcb.200903096 PMC2768842

[bib10] Dunn K. W. Kamocka M. M. McDonald J. H., 2011 A practical guide to evaluating colocalization in biological microscopy. Am. J. Physiol. Cell Physiol. 300: C723–C742. 21209361 10.1152/ajpcell.00462.2010 PMC3074624

[bib11] Fukuda T. Pratto F. Schimenti J. C. Turner J. M. A. Camerini-Otero R. D., 2012 Phosphorylation of chromosome core components may serve as axis marks for the status of chromosomal events during mammalian meiosis. PLoS Genet. 8: e1002485. 22346761 10.1371/journal.pgen.1002485 PMC3276554

[bib12] Fukuda T. Fukuda N. Agostinho A. Hernández‐Hernández A. Kouznetsova A., 2014 STAG3‐mediated stabilization of REC8 cohesin complexes promotes chromosome synapsis during meiosis. EMBO J. 33: 1243–1255. 24797475 10.1002/embj.201387329 PMC4198027

[bib13] Gómez R. Jordan P. W. Viera A. Alsheimer M. Fukuda T., 2013 Dynamic localization of SMC5/6 complex proteins during mammalian meiosis and mitosis implies functions in distinct chromosome processes. J. Cell Sci. 126: 4239–4252. 23843628 10.1242/jcs.130195 PMC3772391

[bib14] Gutiérrez-Caballero C. Herrán Y. Sánchez-Martín M. Suja J. Á. Barbero J. L., 2011 Identification and molecular characterization of the mammalian α-kleisin RAD21L. Cell Cycle 10: 1477–1487. 21527826 10.4161/cc.10.9.15515

[bib15] Handel M. A. Schimenti J. C., 2010 Genetics of mammalian meiosis: regulation, dynamics and impact on fertility. Nat. Rev. Genet. 11: 124–136. 20051984 10.1038/nrg2723

[bib16] Hauf S. Roitinger E. Koch B. Dittrich C. M. Mechtler K., 2005 Dissociation of cohesin from chromosome arms and loss of arm cohesion during early mitosis depends on phosphorylation of SA2. PLoS Biol. 3: e69. 15737063 10.1371/journal.pbio.0030069 PMC1054881

[bib17] Heidmann D. Horn S. Heidmann S. Schleiffer A. Nasmyth K., 2004 The Drosophila meiotic kleisin C(2)M functions before the meiotic divisions. Chromosoma 113: 177–187. 15375666 10.1007/s00412-004-0305-5

[bib18] Herrán Y. Gutierrez-Caballero C. Sanchez-Martin M. Hernandez T. Viera A., 2011 The cohesin subunit RAD21L functions in meiotic synapsis and exhibits sexual dimorphism in fertility. EMBO J. 30: 3091–3105. 21743440 10.1038/emboj.2011.222 PMC3160193

[bib19] Hodges C. A. Revenkova E. Jessberger R. Hassold T. J. Hunt P. A., 2005 SMC1β-deficient female mice provide evidence that cohesins are a missing link in age-related nondisjunction. Nat. Genet. 37: 1351–1355. 16258540 10.1038/ng1672

[bib20] Holzmann J. Fuchs J. Pichler P. Peters J.-M. Mechtler K., 2011 Lesson from the stoichiometry determination of the cohesin complex: a short protease mediated elution increases the recovery from cross-linked antibody-conjugated beads. J Prot. Res. 10: 780–789. 10.1021/pr100927x PMC3033704 21043528

[bib21] Hopkins J. Hwang G. Jacob J. Sapp N. Bedigian R., 2014 Meiosis-specific cohesin component, *Stag3* is essential for maintaining centromere chromatid cohesion, and required for DNA repair and synapsis between homologous chromosomes. PLoS Genet. 10: e1004413. 24992337 10.1371/journal.pgen.1004413 PMC4081007

[bib22] Ishiguro K.-i. Kim J. Fujiyama-Nakamura S. Kato S. Watanabe Y., 2011 A new meiosis-specific cohesin complex implicated in the cohesin code for homologous pairing. EMBO Rep. 12: 267–275. 21274006 10.1038/embor.2011.2 PMC3059921

[bib23] Ishiguro K.-i. Kim J. Shibuya H. Hernández-Hernández A. Suzuki A., 2014 Meiosis-specific cohesin mediates homolog recognition in mouse spermatocytes. Genes Dev. 28: 594–607. 24589552 10.1101/gad.237313.113 PMC3967048

[bib24] Jordan P. W. Karppinen J. Handel M. A., 2012 Polo-like kinase is required for synaptonemal complex disassembly and phosphorylation in mouse spermatocytes. J. Cell Sci. 125: 5061–5072. 22854038 10.1242/jcs.105015 PMC3533391

[bib25] Keeney S. Giroux C. N. Kleckner N., 1997 Meiosis-specific DNA Double-strand breaks are catalyzed by Spo11, a member of a widely conserved protein family. Cell 88: 375–384. 9039264 10.1016/s0092-8674(00)81876-0

[bib26] Khetani R. S. Bickel S. E., 2007 Regulation of meiotic cohesion and chromosome core morphogenesis during pachytene in *Drosophila* oocytes. J. Cell Sci. 120: 3123–3137. 17698920 10.1242/jcs.009977

[bib27] Klein F. Mahr P. Galova M. Buonomo S. B. C. Michaelis C., 1999 A central role for cohesins in sister chromatid cohesion, formation of axial elements, and recombination during yeast meiosis. Cell 98: 91–103. 10412984 10.1016/S0092-8674(00)80609-1

[bib28] La Salle S. Sun F. Handel M. A., 2009 Isolation and short-term culture of mouse spermatocytes for analysis of meiosis. Methods Mol. Biol. 558: 279–297. 19685331 10.1007/978-1-60761-103-5_17

[bib29] Lee J. Hirano T., 2011 RAD21L, a novel cohesin subunit implicated in linking homologous chromosomes in mammalian meiosis. J. Cell Biol. 192: 263–276. 21242291 10.1083/jcb.201008005 PMC3172173

[bib30] Losada A. Yokochi T. Kobayashi R. Hirano T., 2000 Identification and characterization of Sa/Scc3p subunits in the xenopus and human cohesin complexes. J. Cell Biol. 150: 405–416. 10931856 10.1083/jcb.150.3.405 PMC2175199

[bib31] Lin Y.-N. Matzuk M., 2014 Genetics of male fertility. Methods Mol. Biol. 1154: 25–37. 24782004 10.1007/978-1-4939-0659-8_2

[bib32] Llano E. Herrán Y. García-Tuñón I. Gutiérrez-Caballero C. de Álava E., 2012 Meiotic cohesin complexes are essential for the formation of the axial element in mice. J. Cell Biol. 197: 877–885. 22711701 10.1083/jcb.201201100 PMC3384418

[bib33] Llano E. Gomez-H L. García-Tuñón I. Sánchez-Martín M. Caburet S., 2014 STAG3 is a strong candidate gene for male infertility. Hum. Mol. Genet. 23: 3421–3431. 24608227 10.1093/hmg/ddu051

[bib34] Manheim E. A. McKim K. S., 2003 The synaptonemal complex component C(2)M regulates meiotic crossing over in *Drosophila*. Curr. Biol. 13: 276–285. 12593793 10.1016/s0960-9822(03)00050-2

[bib35] Michaelis C. Ciosk R. Nasmyth K., 1997 Cohesins: chromosomal proteins that prevent premature separation of sister chromatids. Cell 91: 35–45. 9335333 10.1016/s0092-8674(01)80007-6

[bib36] Mito Y. Sugimoto A. Yamamoto M., 2003 Distinct developmental function of two *Caenorhabditis elegans* homologs of the cohesin subunit Scc1/Rad21. Mol. Biol. Cell 14: 2399–2409. 12808038 10.1091/mbc.E02-09-0603 PMC194888

[bib37] Moens P. B. Heyting C. Dietrich A. J. van Raamsdonk W. Chen Q., 1987 Synaptonemal complex antigen location and conservation. J. Cell Biol. 105: 93–103. 2440900 10.1083/jcb.105.1.93 PMC2114919

[bib38] Murdoch B. Owen N. Stevense M. Smith H. Nagaoka S., 2013 Altered cohesin gene dosage affects mammalian meiotic chromosome structure and behavior. PLoS Genet. 9: e1003241. 23408896 10.1371/journal.pgen.1003241 PMC3567145

[bib39] Nasmyth K. Haering C. H., 2005 The structure and function of SMC and kleisin complexes. Annu. Rev. Biochem. 74: 595–648. 15952899 10.1146/annurev.biochem.74.082803.133219

[bib40] Novak I. Wang H. Revenkova E. Jessberger R. Scherthan H., 2008 Cohesin SMC1β determines meiotic chromatin axis loop organization. J. Cell Biol. 180: 83–90. 18180366 10.1083/jcb.200706136 PMC2213612

[bib41] Offenberg H. H. Schalk J. A. C. Meuwissen R. L. J. van Aalderen M. Kester H. A., 1998 SCP2: A major protein component of the axial elements of synaptonemal complexes of the rat. Nucleic Acids Res. 26: 2572–2579. 9592139 10.1093/nar/26.11.2572 PMC147596

[bib42] Orgil O. Matityahu A. Eng T. Guacci V. Koshland D., 2015 A conserved domain in the Scc3 subunit of cohesin mediates the interaction with both Mcd1 and the cohesin loader complex. PLoS Genet. 11: e1005036. 25748820 10.1371/journal.pgen.1005036 PMC4352044

[bib43] Pasierbek P. Födermayr M. Jantsch V. Jantsch M. Schweizer D., 2003 The *Caenorhabditis elegans* SCC-3 homologue is required for meiotic synapsis and for proper chromosome disjunction in mitosis and meiosis. Exp. Cell Res. 289: 245–255. 14499625 10.1016/s0014-4827(03)00266-0

[bib44] Phadnis N. Cipak L. Polakova S. Hyppa R. W. Cipakova I., 2015 Casein kinase 1 and phosphorylation of cohesin subunit Rec11 (SA3) promote meiotic recombination through linear element formation. PLoS Genet. 11: e1005225. 25993311 10.1371/journal.pgen.1005225 PMC4439085

[bib45] Prieto I. Suja J. A. Pezzi N. Kremer L. Martinez-A C., 2001 Mammalian STAG3 is a cohesin specific to sister chromatid arms in meiosis I. Nat. Cell Biol. 3: 761–766. 11483963 10.1038/35087082

[bib61] Prieto I. Pezzi N. Buesa J. M. Kremer L. Barthelemy I., 2002 STAG2 and Rad21 mammalian mitotic cohesins are implicated in meiosis. EMBO Rep. 3: 543–550. 12034751 10.1093/embo-reports/kvf108 PMC1084142

[bib46] Revenkova E. Eijpe M. Heyting C. Hodges C. A. Hunt P. A., 2004 Cohesin SMC1β is required for meiotic chromosome dynamics, sister chromatid cohesion and DNA recombination. Nat. Cell Biol. 6: 555–562. 15146193 10.1038/ncb1135

[bib47] Roig M. B. Löwe J. Chan K.-L. Beckouët F. Metson J., 2014 Structure and function of cohesin’s Scc3/SA regulatory subunit. FEBS Lett. 588: 3692–3702. 25171859 10.1016/j.febslet.2014.08.015 PMC4175184

[bib48] Rowland B. D. Roig M. B. Nishino T. Kurze A. Uluocak P., 2009 Building sister chromatid cohesion: Smc3 acetylation counteracts an antiestablishment activity. Mol. Cell 33: 763–774. 19328069 10.1016/j.molcel.2009.02.028

[bib49] Royo H. Prosser H. Ruzankina Y. Mahadevaiah S. K. Cloutier J. M., 2013 ATR acts stage specifically to regulate multiple aspects of mammalian meiotic silencing. Genes Dev. 27: 1484–1494. 23824539 10.1101/gad.219477.113 PMC3713429

[bib50] Sakuno T. Watanabe Y., 2015 Phosphorylation of cohesin Rec11/SA3 by casein kinase 1 promotes homologous recombination by assembling the meiotic chromosome axis. Dev. Cell 32: 220–230. 25579976 10.1016/j.devcel.2014.11.033

[bib51] Scherthan H. Weich S. Schwegler H. Heyting C. Härle M., 1996 Centromere and telomere movements during early meiotic prophase of mouse and man are associated with the onset of chromosome pairing. J. Cell Biol. 134: 1109–1125. 8794855 10.1083/jcb.134.5.1109 PMC2120985

[bib52] Shibuya H. Watanabe Y., 2014 The meiosis-specific modification of mammalian telomeres. Cell Cycle 13: 2024–2028. 24870409 10.4161/cc.29350 PMC4111693

[bib53] Shibuya H. Ishiguro K.-i. Watanabe Y., 2014 The TRF1-binding protein TERB1 promotes chromosome movement and telomere rigidity in meiosis. Nat. Cell Biol. 16: 145–156. 24413433 10.1038/ncb2896

[bib54] Skarnes W. C. Rosen B. West A. P. Koutsourakis M. Bushell W., 2011 A conditional knockout resource for the genome-wide study of mouse gene function. Nature 474: 337–342. 21677750 10.1038/nature10163 PMC3572410

[bib55] Sumara I. Vorlaufer E. Gieffers C. Peters B. H. Peters J.-M., 2000 Characterization of vertebrate cohesin complexes and their regulation in prophase. J. Cell Biol. 151: 749–762. 11076961 10.1083/jcb.151.4.749 PMC2169443

[bib56] Sutani T. Kawaguchi T. Kanno R. Itoh T. Shirahige K., 2009 Budding yeast Wpl1(Rad61)-Pds5 complex counteracts sister chromatid cohesion-establishing reaction. Curr. Biol. 19: 492–497. 19268589 10.1016/j.cub.2009.01.062

[bib57] Tóth A. Ciosk R. Uhlmann F. Galova M. Schleiffer A., 1999 Yeast cohesin complex requires a conserved protein, Eco1p(Ctf7), to establish cohesion between sister chromatids during DNA replication. Genes Dev. 13: 320–333. 9990856 10.1101/gad.13.3.320 PMC316435

[bib58] Winters T. McNicoll F. Jessberger R., 2014 Meiotic cohesin STAG3 is required for chromosome axis formation and sister chromatid cohesion. EMBO J. 33: 1256–1270. 24797474 10.1002/embj.201387330 PMC4198028

[bib59] Xu H. Beasley M. D. Warren W. D. van der Horst G. T. McKay M. J., 2005 Absence of mouse REC8 cohesin promotes synapsis of sister chromatids in meiosis. Dev. Cell 8: 949–961. 15935783 10.1016/j.devcel.2005.03.018

[bib60] Yuan L. Liu J.-G. Zhao J. Brundell E. Daneholt B., 2000 The murine SCP3 gene is required for synaptonemal complex assembly, chromosome synapsis, and male fertility. Mol. Cell 5: 73–83. 10678170 10.1016/s1097-2765(00)80404-9

