# Supplementary material for: Genetic Interactions Between the Meiosis-Specific Cohesin Components, STAG3, REC8, and RAD21L
Source: G3 (Bethesda). 2016 Apr 16;6(6):1713–24. doi: 10.1534/g3.116.029462 (PMC4889667; doi:10.1534/g3.116.029462)
Supplement: Supplemental Material [file supp_6_6_1713__index.html]

Genetic Interactions Between the Meiosis-Specific Cohesin Components, STAG3, REC8, and RAD21L — Supplemental Material 

# Genetic Interactions Between the Meiosis-Specific Cohesin Components, STAG3, REC8, and RAD21L

## Supplemental Material for Ward *et al.*, 2016

**Files in this Data Supplement:**

- Figure S1 - *Rad21l* mutant allele used in this study. (.pdf, 144 KB)
- Figure S2 - Cohesin mutants result in reduced testis size due to early prophase I arrest. (.pdf, 479 KB)
- Figure S3 - Combining the *Stag3JAX* mutation with *Rec8* or *Rad21l* mutations result in decreased axis length and increased axis number. (.pdf, 455 KB)
- Figure S4 - The *Stag3JAX* mutation supports the hypothesis that STAG3 maintains centromere cohesion, which is primarily mediated by REC8-containing cohesin complexes. (.pdf, 511 KB)
- Figure S5 - Method used for counting pericentromeric heterochromatin clusters (chromocenters). (.pdf, 300 KB)
- Figure S6 - STAG1 and STAG2 localization on chromosome spreads from wild-type and *Stag3OV* knockout primary spermatocytes. (.pdf, 163 KB)
- Table S1 - Antibodies used for immunofluorescence microscopy analyses. (.pdf, 410 KB)
