# Supplementary material for: Genetic Interactions Between the Meiosis-Specific Cohesin Components, STAG3, REC8, and RAD21L
Source: G3 (Bethesda). 2016 Apr 16;6(6):1713–24. doi: 10.1534/g3.116.029462 (PMC4889667; doi:10.1534/g3.116.029462)
Supplement: Supplemental Material [file supp_g3.116.029462_FigureS1.pdf]

Rad21l mutation – JAX allele

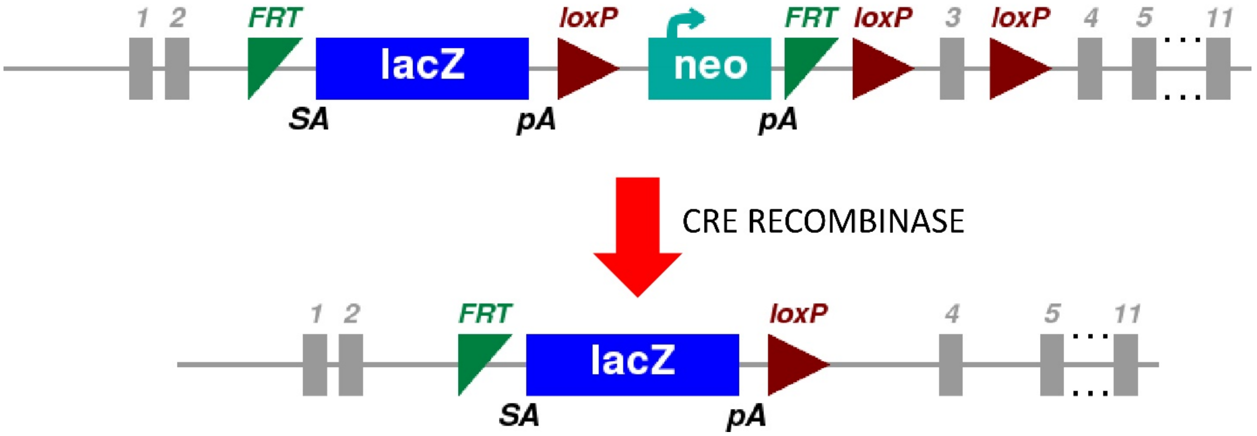

Figure S1: *Rad21l* mutant allele used in this study. The *Rad21l*^{JAX} allele C57BL/6N-derived JM8.N4 embryonic stem (ES) cells that were targeted with a *β-galactosidase* containing cassette that generated a knockout first reporter allele for *Rad21l* that harbored a floxed exon 3 were sourced from the International Knockout Mouse Consortium (Skarnes *et al.* 2011), http://www.knockoutmouse.org/ martsearch/project/22907). As part of the KOMP2 program (http://commonfund.nih.gov/KOMP2/), these ES cells were injected into B6(Cg)-Tyrc-2J/J blastocysts. The resulting chimeric males were bred to C57BL/6NJ females and then to B6N.Cg-Tg(Sox2-cre)1Amc/J mice to excise the floxed neomycin cassette and exon 3, resulting in the heterozygote B6N(Cg)-Rad21ltm1b(KOMP)Wtsi/2J strain used in this study. The *Stag3* and *Rec8* mutant alleles used in this study have been previously described (Bannister *et al.* 2004; Hopkins *et al.* 2014).
