# Supplementary material for: Genetic Interactions Between the Meiosis-Specific Cohesin Components, STAG3, REC8, and RAD21L
Source: G3 (Bethesda). 2016 Apr 16;6(6):1713–24. doi: 10.1534/g3.116.029462 (PMC4889667; doi:10.1534/g3.116.029462)
Supplement: Supplemental Material [file supp_g3.116.029462_FigureS2.pdf]

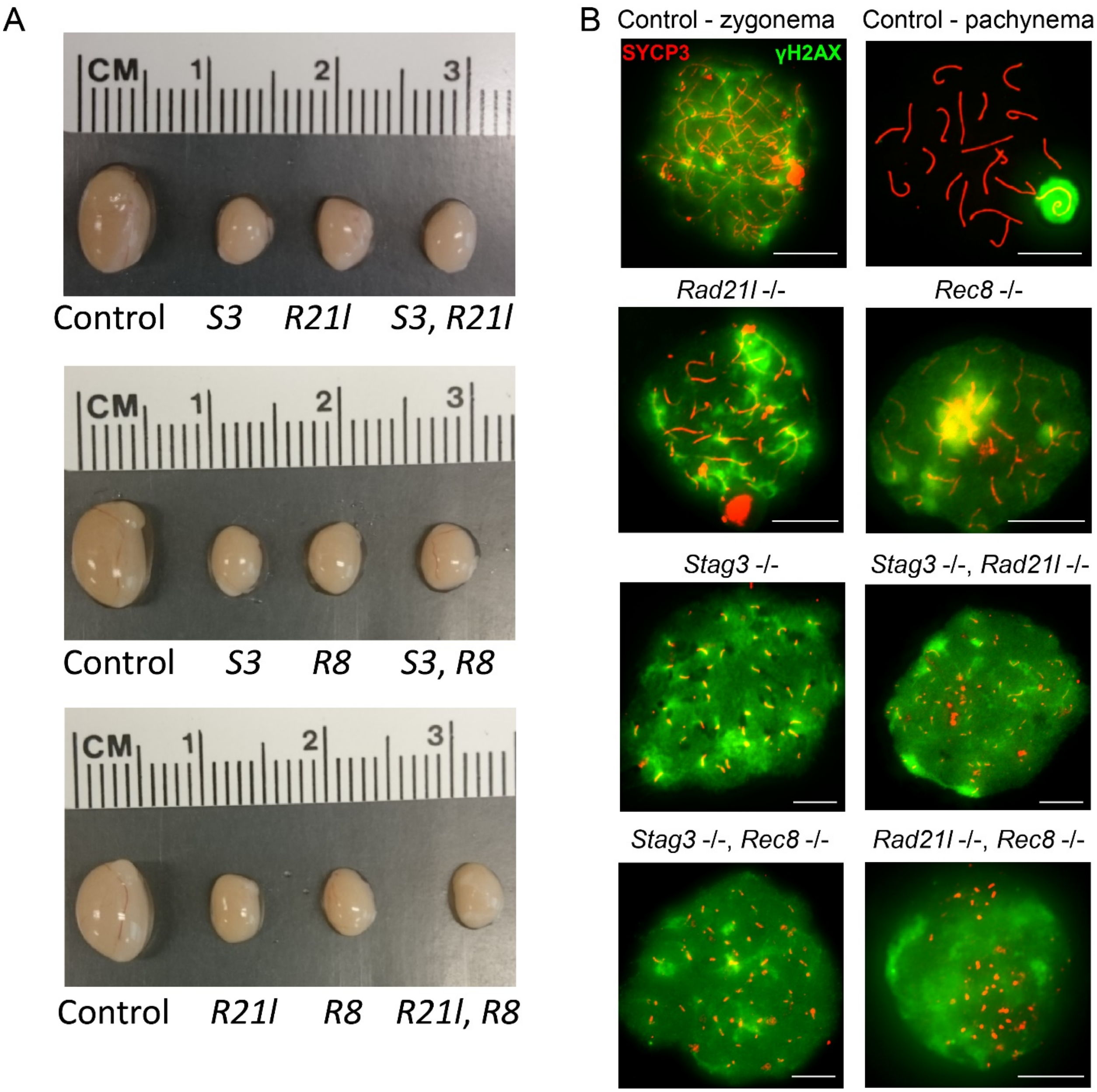

Figure S2: Cohesin mutants result in reduced testis size due to early prophase I arrest. (A) Testis size comparison between control and mutant testes extracted from adult mice, 8-12 weeks post-partum (*S3* = *Stag3*, *R21l* = *Rad21l* and *R8* = *Rec8*). (B) Example chromatin spread preparations from purified testicular germ cells of control, *Rad21l*, *Rec8*, *Stag3* single mutants and the three possible double mutant combinations aged 15 days post-partum. Chromatin spreads were immunolabeled using antibodies against the SC lateral element protein SYCP3 (red) and γH2AX (green). Zygotene and pachytene stages are depicted for control, and typical examples for each mutant are given. Scale bars = 10 μm.
