# Supplementary material for: Genetic Interactions Between the Meiosis-Specific Cohesin Components, STAG3, REC8, and RAD21L
Source: G3 (Bethesda). 2016 Apr 16;6(6):1713–24. doi: 10.1534/g3.116.029462 (PMC4889667; doi:10.1534/g3.116.029462)
Supplement: Supplemental Material [file supp_g3.116.029462_FigureS3.pdf]

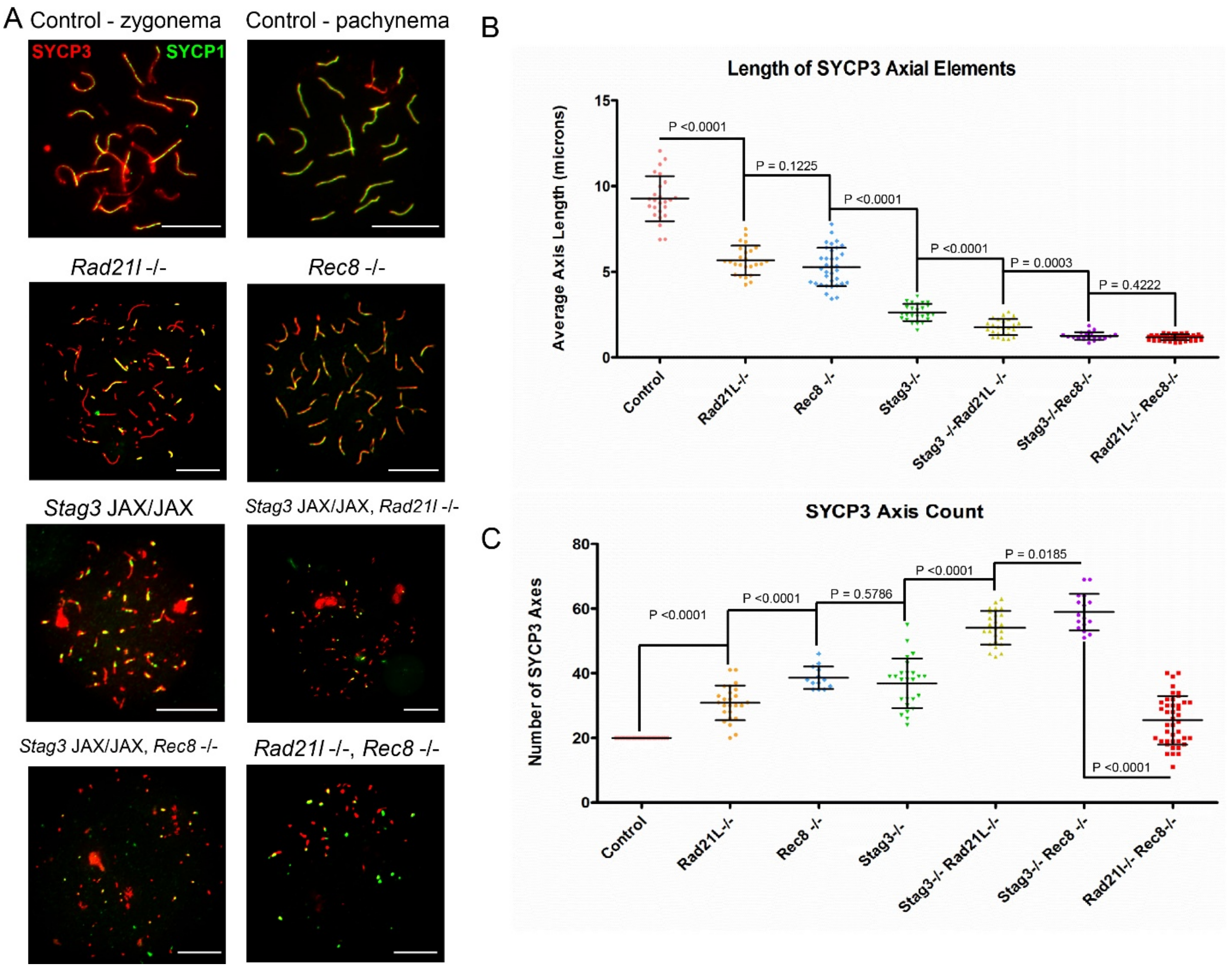

Figure S3: Combining the *Stag3*^{JAX} mutation with *Rec8* or *Rad21l* mutations result in decreased axis length and increased axis number. (A) Example chromatin spread preparations from purified testicular germ cells of control, *Rad21l*, *Rec8*, *Stag3* single mutants and the three possible double mutant combinations aged 15 days post-partum. Chromatin spreads were immunolabeled using antibodies against the SC lateral element protein SYCP3 (red) and the transverse filament of the central region of the SC SYCP1 (green). Zygotene and pachytene stages are depicted for control, and typical examples for each mutant are given. (B) Scatter dot-plot graph of the average SYCP3 length per spermatocyte chromatin spread. (C) Scatter dot-plot graph of the number of SYCP3 linear stretches per spermatocyte chromatin spread. Mean and standard deviation of the columns of each graph are represented by the black bars and *P* values are given for indicated comparisons (Mann-Whitney, two-tailed), significant differences were defined when the P value was < 0.05 otherwise it was considered not significant. Experiments were performed using 2 separate littermate pairs of mutant and control mice. Scale bars = 10 µm.
