# Supplementary material for: Genetic Interactions Between the Meiosis-Specific Cohesin Components, STAG3, REC8, and RAD21L
Source: G3 (Bethesda). 2016 Apr 16;6(6):1713–24. doi: 10.1534/g3.116.029462 (PMC4889667; doi:10.1534/g3.116.029462)
Supplement: Supplemental Material [file supp_g3.116.029462_FigureS4.pdf]

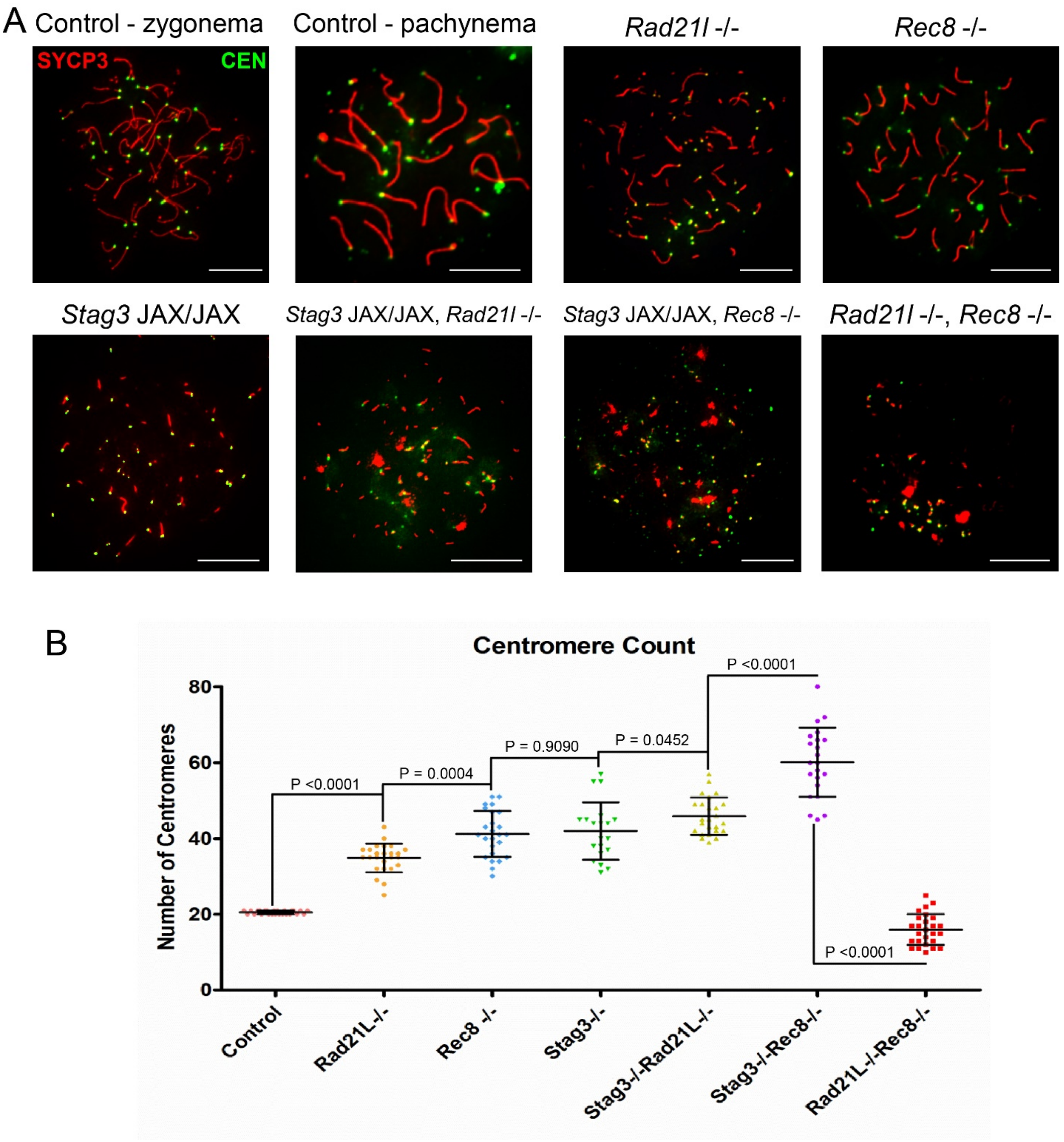

Figure S4: The *Stag3*^{JAX} mutation supports the hypothesis that STAG3 maintains centromere cohesion, which is primarily mediated by REC8-containing cohesin complexes. (A) Example chromatin spread preparations from purified testicular germ cells of control, *Rad21l*, *Rec8*, *Stag3* single mutants and the three possible double mutant combinations aged 15 days post-partum. Chromatin spreads were immunolabeled using antibodies against the SC lateral element protein SYCP3 (red) and the CEN anti-centromere autoantibody (green). Zygotene and pachytene stages are depicted for control, and typical examples for each mutant are given. (B) Scatter dot-plot graph

of the average number of centromere signals per spermatocyte chromatin spread. Mean and standard deviation of the columns of each graph are represented by the black bars and *P* values are given for indicated comparisons (Mann-Whitney, two-tailed), significant differences were defined when the P value was < 0.05 otherwise it was considered not significant. Experiments were performed using 2 separate littermate pairs of mutant and control mice. Scale bars = 10 µm.
