# Supplementary material for: Genetic Interactions Between the Meiosis-Specific Cohesin Components, STAG3, REC8, and RAD21L
Source: G3 (Bethesda). 2016 Apr 16;6(6):1713–24. doi: 10.1534/g3.116.029462 (PMC4889667; doi:10.1534/g3.116.029462)
Supplement: Supplemental Material [file supp_g3.116.029462_FigureS5.pdf]

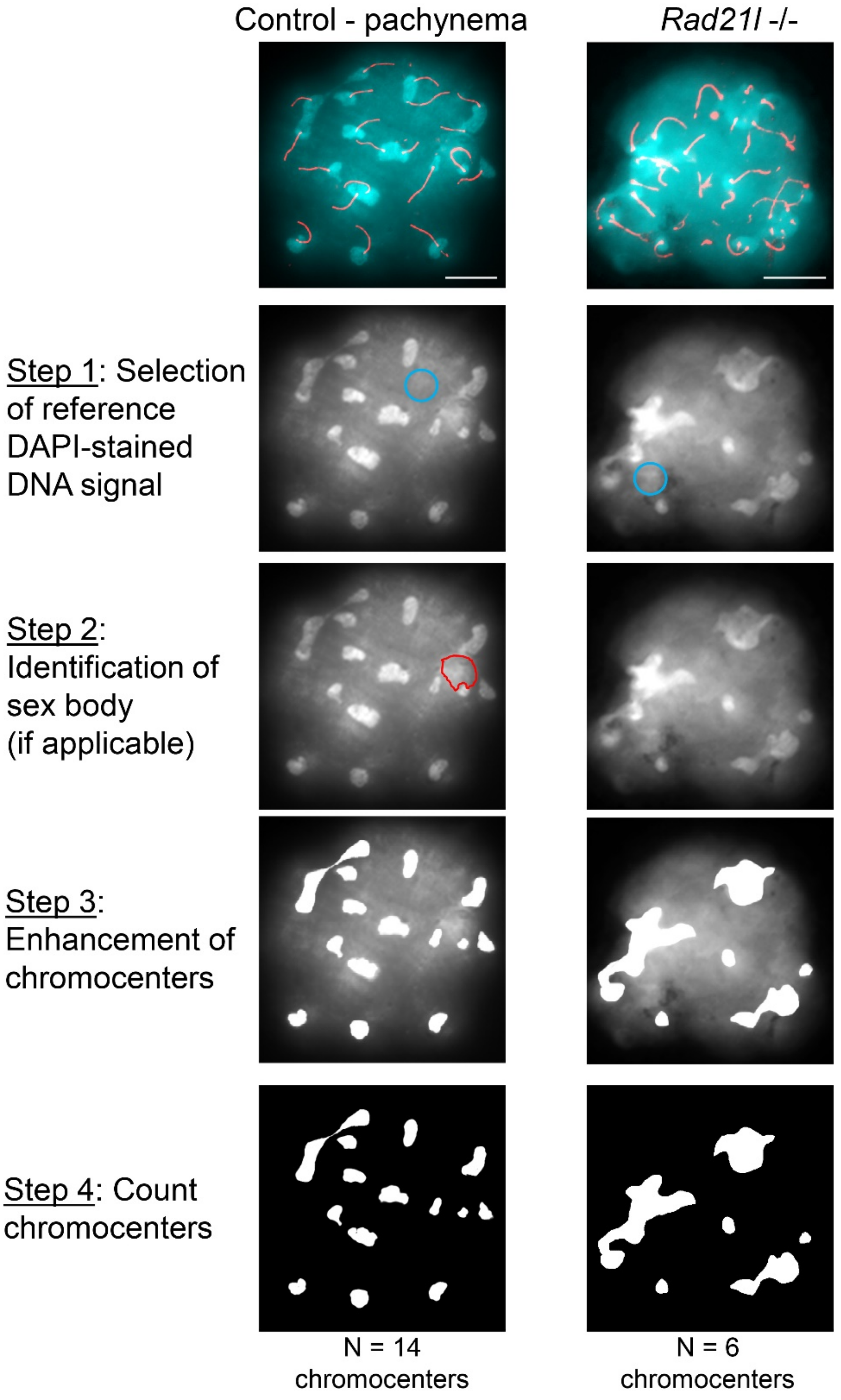

Figure S5: Method used for counting pericentromeric heterochromatin clusters (chromocenters). Each image was captured with the same exposure time. We developed a procedure using image J that follows these steps: 1) Optimize image contrast, and select the highest intensity of the non-chromocenter DAPI stain signal (blue dotted ring). 2) The sex body can then be selected to ensure it is not included in the analysis (red dotted ring). 3) Signals above the selected DAPI signal in

step 1 are maximized. 4) The remaining DAPI signal is then removed and the chromocenter signals can be counted. Scale bars = 10 µm.
