# Supplementary material for: Genetic Interactions Between the Meiosis-Specific Cohesin Components, STAG3, REC8, and RAD21L
Source: G3 (Bethesda). 2016 Apr 16;6(6):1713–24. doi: 10.1534/g3.116.029462 (PMC4889667; doi:10.1534/g3.116.029462)
Supplement: Supplemental Material [file supp_g3.116.029462_FigureS6.pdf]

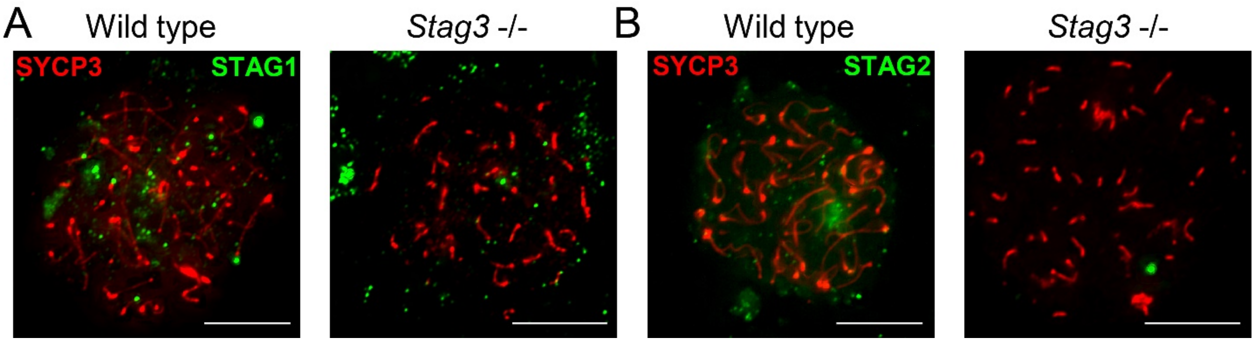

Figure S6: STAG1 and STAG2 localization on chromosome spreads from wild-type and *Stag3*^{OV} knockout primary spermatocytes. (A) Example chromatin spread preparations immunolabeled using antibodies against the SC lateral element protein SYCP3 (red) and the STAG1 (green). (B) Example chromatin spread preparations immunolabeled using antibodies against the SC lateral element protein SYCP3 (red) and the STAG2 (green).
