# Supplementary material for: Genetic Interactions Between the Meiosis-Specific Cohesin Components, STAG3, REC8, and RAD21L
Source: G3 (Bethesda). 2016 Apr 16;6(6):1713–24. doi: 10.1534/g3.116.029462 (PMC4889667; doi:10.1534/g3.116.029462)
Supplement: Supplemental Material [file supp_g3.116.029462_TableS1.pdf]

Table S1. Antibodies used for immunofluorescence microscopy analyses.

Antigen	Host	Source	Catalogue number	Dilution ratio
CREST/ACA (CEN)	Human	Antibodies Incorporated	15-235	1:50
RAD21	Rabbit	Abcam	ab154769	1:250
RAD21L	Rabbit	Alberto Pendas	NA	1:250
REC8	Rabbit	Karen Schindler	NA	1:500
SMC1α	Rabbit	Rolf Jessberger	NA	1:100
SMC1β	Rabbit	Rolf Jessberger	NA	1:250
SMC3	Rabbit	Abcam	ab9263	1:250
STAG1	Goat	Abcam	ab4457	1:25
STAG2	Goat	Abcam	ab4463	1:25
SYCP1	Rabbit	Novus Biologicals	NB300-229	1:500
SYCP3	Rat	Mary Ann Handel	NA	1:500
SYCP3	Mouse	Santa Cruz	Sc-74569	1:50
γH2AX	Mouse	Millipore	05-636	1:500
